# Artificial neuro-fuzzy intelligence for dynamic tracking and prediction of HIV prevalence and new infections among individuals aged 15 to 49 years in Uganda: Computational national-level models

**DOI:** 10.1097/MD.0000000000050760

**Published:** 2026-09-18

**Authors:** Elnazeer Ali Hamid Abdalla, Muna Elsadig, Umi Omar, Val Hyginus Udoka Eze, Adil Mergani, Amina Salhi, Abuagla M. Dafalla, Yasreen Gasm Alkhalig, Sahla Mohammed Ahmed, Bienfait Mumbere Vahwere

**Affiliations:** a Department of Electrical, Telecommunication and Computer Engineering, Kampala International University, Ishaka, Uganda; b Department of Information Systems, College of Computer and Information Sciences, Princess Nourah bint Abdulrahman University, Riyadh, Saudi Arabia; c CEO of Kampala International University Teaching Hospital and Research, Ishaka, Uganda; d Department of Electrical and Electronics Engineering, Mbarara University of Science and Technology, Kihumuro, Uganda; e Department of Molecular Biology and Genetics, Kampala International University, Ishaka, Uganda; f School of Allied Health Sciences, Kampala International University, Ishaka, Uganda; g School of Architecture, Computing, and Engineering, University of East London, London United Kingdom; h Department of Physiology, Kampala International University, Ishaka, Uganda; i Department of Surgery, Kampala International University, Ishaka, Uganda.

**Keywords:** AI-based ANFIS, GNN, HIV prevalence, new HIV infection, people living with HIV

## Abstract

Human immunodeficiency virus (HIV) infection remains a major global health burden, and Uganda continues to experience rising new HIV incidence, making prevention and management increasingly challenging. Accurate estimation of HIV prevalence (PHIVP) and tracking new HIV incidence (PHIVN) require robust computational models to effectively inform public health interventions. This study aims to develop a computational tracking model based on a 2-level intelligent framework to dynamically monitor PHIVP and PHIVN in Uganda and to identify influential factors that contribute to HIV trends among individuals aged 15 to 49. Six datasets from 146 Ugandan districts for 2023: sex rate, antiretroviral therapy coverage, awareness, education, personal income, and historical HIV indicators (prevalence and new incidence) were analyzed. A 2-level intelligent technique was employed: level I, an upgraded clustering algorithm was used to identify optimal parameters influencing PHIVP and PHIVN and level II, a neuro-fuzzy model was trained on these parameters to construct the computational tracking model. Data were partitioned into 85% for model training and 15% for testing and validation. Model accuracy was evaluated using regression performance, error metrics, and statistical significance. The model predicted a decline in PHIVP from 5.1% to 4.92%, and accurately tracked 37,996 PHIVN cases out of 38,132 actual incidences. The computational framework demonstrated strong predictive performance, yielding high regression values of *R* = 0.832 to 0.987 for PHIVP and PHIVN across 3 scenarios. Statistical analysis confirmed model reliability with *P* < .001 and *P* = .001. The 2-level intelligent approach exhibits strong potential for dynamically monitoring HIV prevalence and new incidence in Uganda. The 6 analyzed datasets represent significant determinants of PHIVP reduction and PHIVN tracking among adults aged 15 to 49. These findings support the feasibility of computational modeling as a tool for enhancing HIV surveillance, guiding prevention strategies, and strengthening evidence-based public health decision-making in Uganda.

## 1. Introduction

According to the Uganda Ministry of Health’s 2023 estimates, approximately 1492,407 people are living with human immunodeficiency virus (HIV) (PLHIV – all ages), of which approximately 1.4 million are aged 15 to 49 years https://www.mnypa.org/docs/1723095739.pdf. HIV remains a major public health concern in Uganda due to persistent challenges such as sexual activity, economic inequality, and limited access to sexual health education.^[1]^ HIV prevalence control efforts in Uganda are hampered by several challenges, including multiple sexual partners, poverty, which increases an individual’s susceptibility to HIV, and knowledge gaps due to a lack of sex education.^[1]^ Also, a lack of access to healthcare facilities for antiretroviral therapy (ART) therapy, as well as a lack of affordability, knowledge, and education, are the main causes of the rising prevalence of HIV. However, over the past 15 years, Uganda has achieved tremendous progress in addressing the HIV decline using medical therapy-based ART.

Despite a decline in HIV prevalence (PHIVP) from 7.2% to 5.1% and new HIV incidence (PHIVN) from 69,000 to 33,000 cases (https://www.mnypa.org/docs/1723095739.pdf). The reduction in PHIVP and PHIVN has been largely attributed to ART, pre-exposure prophylaxis, awareness, and socioeconomic improvements.^[2–7]^ While Uganda has implemented some preventative strategies to reduce HIV prevalence and new infections,^[1,8]^ unsafe sex and multiple sexual behaviors continue to pose a serious risk to PLHIV (15–49 years). Numerous constraints pertaining to the data collection procedure and other systematic challenges limit the HIV statistics in Uganda that use numerical algorithms, hybrid prevalence estimates, and other regressions.^[9,10]^

### 1.1. Literature review: similar works

Artificial intelligence (AI) and machine learning (ML) offer promising applications in HIV response, including treatment planning, testing, early diagnosis, prediction, and monitoring of prevalence trends.^[11–15]^ Considering awareness and infection transmission knowledge, studies have demonstrated the potential of AI/ML in modeling HIV dynamics, optimizing ART dosing, and predicting infection patterns.^[12,16–18]^ For example, national-level modeling consortia have developed tools to predict HIV prevalence, ART coverage, and stigma indices, whereas others have applied graph neural networks (GNNs) and empirical models for incidence prediction and prevalence estimation. District-level modeling systems have also been implemented to support diagnostic procedures and intervention planning.^[19–21]^ Global studies further highlight variations in HIV prevalence by age, sex, and region, with the highest burden observed among PLHIV aged 25 to 54 years. Statistical models have been used to quantify gaps in ART coverage and vertical transmission prevention, whereas other models address virologic failure risks among adolescents by incorporating sociodemographic, behavioral, and economic factors. Analytical frameworks have additionally estimated prevalence and incidence based on ART status, mortality, and demographic profiles.^[18,22–24]^ Furthermore, multiple models have been formulated, aiming to estimate and predict new cases of HIV and its prevalence among PLHIV aged 15 to 49 years based on key populations.^[25]^ In contrast, a number of systematic and analytical studies have been conducted to examine immunological and risk variables impacting HIV prevalence and new infections among PLHIV.^[26–29]^ A study in^[30]^ developed a prediction model to control the risk of virologic failure associated with HIV in teenagers. Treatment, sociodemographic, behavioral, psychological, and economic factors are among the variables that the model uses to control the exposure of anonymous and distinct HIV adolescents. Also, in analytical studies,^[31,32]^ the rates of HIV prevalence and incidence of new HIV infections have been estimated using age and sex as well as ART therapy. Studies^[33,34]^ projected the PHIVP and PHIVN using different mathematical models. Table 1 summarizes the PHIVP trend, including suggested methods, objectives, key findings, and a call to action.

**Table 1:** An overview of HIV incidence and prevalence using various approaches.

Date	Ref.	Method	Objective	Key findings	An urgent call to action
2019	^[35]^	Developed PLHIV resilience scale as stigma index	The aim of this study is to assess PLHIV’s quality of life.	Assisting in interventions and policies information. The results indicated lower resilience scales and greater depression ratings.	More studies are needed to encompass all countries affected by HIV to enhance the lives of PLHIV based on the stigma index.
2024	^[36]^	A cross-sectional analysis case-study conducted at Kiruddu National Referral Hospital	This study analyzes and optimizes the factors that help in treatment and care of PLHIV in hospitals	The study demonstrated that the predicted factors related to PLHIV and other illnesses that affect Uganda’s death rate play a key role in managing patient care and treatment	For the study analysis to be successful, additional variables, such as the sex gender ratio must be provided.
2023	^[23]^	A systematic analysis has developed-based HIV/AIDS data collection	The aim of the case study is to track global HIV/AIDS prevalence statistics in order to evaluate risk variables and death rates by age, sex, and geography.	When it comes to intimacy (sex rate), the HIV prevalence burden is larger in a region with a high sociodemographic index, both in terms of temporal and spatial trends. HIV prevalence and infection rates are greater in the 25– 54 age range.	More analytical research that takes into account awareness, education, and full funding is required. Additionally, planned action through decision-making that needs to be put into action immediately is needed to prevent HIV/AIDS prevalence.
2024	^[37]^	Integrated DHS data were used to create a multiple logistic regression for 8 countries.	This study aims to ascertain the frequency of discriminatory attitudes toward patients with HIV/AIDS and the associated factors that are linked to these attitudes among women	Due to poverty and low levels of education, more than half of the women did not utilize any form of birth control. The study found that women’s perceptions about HIV/AIDS continued to be very discriminating. Additionally, it showed that women living with HIV between the ages of 15 and 24 have a greater rate in comparison to other groups because they have a prejudiced attitude toward HIV/AIDS.	A call to action is required to combat HIV prejudice against women. Therefore, there must be no stigma, and that can only be achieved by education, awareness, and community.
2024	^[24]^	Spectrum/EPP/Naomi model that is used to estimate HIV prevalence nationally/sub-nationally	The study targets to evaluate coverage gaps in HIV prevalence initiatives such as ART medication and vertical transmission prevention	This method-based data targeted sub-national planning approach for HIV prevention. It has been prioritizing regions and populations and recommended an annual evaluation study.	Pregnant women in Kenya have a high cumulative percentage of positive HIV on ART, and the country has the highest rate of new HIV infections. In order to follow any new HIV infection, an urgent call must be made every 6 mo, particularly for women who tested positive for the virus while receiving ART.
2024	^[25]^	A new incidence-based multiple model has been used for HIV prevalence in those PLHIV who aged 15–49 between 2010 and 2022.	The study aims to estimate and predict the new incidence of HIV, and then a priority action plan will take place.	The model estimates lower proportions of new incidence among key populations compared to the report (2016–2022), which was published by UNAIDS.	The study indicates that the number of new HIV infections increases among key populations. This study will pave the way for the development of additional multiple models for classifying all aged groups.
2024	^[21]^	Graph neural network (GNN)	The goal of this study-based data is to predict and estimate HIV infection in order to reduce its prevalence.	The findings showed a good performance using a data of 2 cities	It should be highlighted that the study lacks validation datasets to back up its conclusions. Not every feature in the data can be quantified by the GNN alone. An algorithm can be combined with GNN to quantify every feature.
2024	^[38]^	Fractional model and optimum control measures	This study aims to develop an optimal model to identify and control the exposed and unidentified individuals with HIV.	The model is considered a helpful tool for exposing HIV-infected people, and it demonstrated a good performance.	The model needs new parameters and an updated numerical method to identify any new potential HIV cases, whether through violence, STDs, or other ways.
2025	^[30]^	Estimated model: for adolescents living with HIV using sociodemographic, behavioral, psychological, economic, and treatment factors.	The main aim of this study is to predict the HIV risk of virologic failure among adolescents aged 10–16.	The prediction model showed promise in identifying people who are more likely to experience virologic failure.	There is a **need** for predictive models to identify virologic failure in individual groups for all ages.

AIDS = acquired immune deficiency syndrome, ART = antiretroviral therapy, DHS = demographic and health surveys, EPP = estimation and projection package, GNN = graph neural network, HIV = human immunodeficiency virus, PHIVN = new HIV incidence, PHIVP = HIV prevalence, STDs = sexually transmitted diseases.

Although statistical and deterministic compartmental models often fail to capture nonlinear data, stratified cluster sampling model-based spectrum and 2 more robust techniques have been employed in Uganda’s population-based HIV impact assessment.^[8,18,39–42]^ Their limitations have become increasingly prominent when processing PHIVP and PHIVN load data modeling. The projection of PHIVP and PHIVN data contains “seasonal (peak and valley) data clustering” driven by associated HIV factors. However, the status awareness and ART factors are used to project the statistical models for PHIVP and PHIVN in the district/population-level-based survey utilizing stratified cluster, district health information software, estimation and projection package/spectrum, and *prevR* method.^[42–44]^ These are restricted to estimating small areas, and it was found that the methods are unreliable and imprecise due to the uncertainty surrounding the validity of HIV estimates in specific districts or regions. Despite using GNN (as an AI) to reduce HIV prevalence, it does not include validation datasets to support its conclusions.^[21]^ To obtain accurate and trustworthy HIV statistics in Uganda, these limitations underscore the effectiveness of continuous efforts to improve data quality assurance and enhance data training for workers. Nevertheless, accurate prediction and tracking models are essential for informing public health policies and optimizing interventions.^[18,45,46]^ The application of AI-based adaptive neuro-fuzzy inference systems (ANFIS) models can capture nonlinear data effectively; however, there is no AI-based ANFIS study in Uganda to address HIV prevalence and new incidence, despite its success in epidemiology and healthcare. This study aimed to develop a novel AI-based prediction model to track and estimate PHIVP) and PHIVN in Uganda, with a focus on supporting evidence-based strategies for sustaining control measures and enhancing national response efforts.

This research work intends to fill these research gaps by developing a dynamic ANFIS-based upgraded clustering algorithm for tracking PHIVP and PHIVN in 146 Ugandan districts based on 2023 historical data. This research, therefore, has the intention of making a scientific contribution with a significant impact on HIV prevalence and new infections. The major contributions of this study are summarized as follows:

- Development of a novel fuzzy subtractive clustering (FSC)–accelerated particle swarm optimization (APSO)–ANFIS framework that integrates the evolutionary symbolic learning capability of upgraded clustering (FSC–APSO) with the adaptive nonlinear inference strength of ANFIS, addressing a critical gap in PHIVP and PHIVN prediction studies.
- Comparative benchmarking against advanced AI, including FSC–ANFIS and FSC–APSO–ANFIS, with existing 2023 Uganda data using performance metrics (root mean square error [RMSE], regression, and model reliability).
- Provision of a reliable technique approach offering dynamic tracking of PLHIV to improve and reduce the spread of HIV and its incidence in comparison with GNN and enhanced ANFIS models found in Refs.^[21]^ and^[47]^ respectively, as well as the 2023 statistical data obtained from (https://www.mnypa.org/docs/1723095739.pdf)

This projection model’s study contributes to the body of knowledge by providing associated factors that can assist in identifying the lowest and highest HIV incidences and projecting the overall prevalence percentage in Uganda. The findings of tracking HIV are significant for allocating resources by optimizing ART, increasing awareness, and maximizing educational tools to enable decision-makers to create an immediate action plan.

### 1.2. PLHIV action plan in Uganda

Young people, men, parents, leaders, and the community were urged by the Ugandan government and the Uganda acquired immune deficiency syndrome (AIDS) Commission (https://www.mnypa.org/docs/1723095739.pdf) to take immediate action to reduce the spread of HIV infection and lower the number of PLHIV by implementing the following sustainable measures:

1.2.1. Using condoms when having sex.
1.2.2. Education, awareness-raising, and providing free ART to people living with HIV.
1.2.3. Newborns must be separated from their mothers, and breastfeeding should be declined.
1.2.4. Safe male circumcision provides significant protection against HIV infections.
1.2.5. Stopping HIV-related oral lesions, defilement, and domestic violence.

The increase in family awareness (FMA), educational level (EDU), and individual income rate (INC) has a positive impact on the decline of PLHIV. Moreover, treatment via ART is essential to prevent HIV transmission from mother to child. To track overall PHIVP and identify PHIVN, the proposed model would be carried out in 3 phases. These phases include data collection and identification, data optimization, and data prediction.

## 2. Methods

### 2.1. Overview

**Figure 1.**
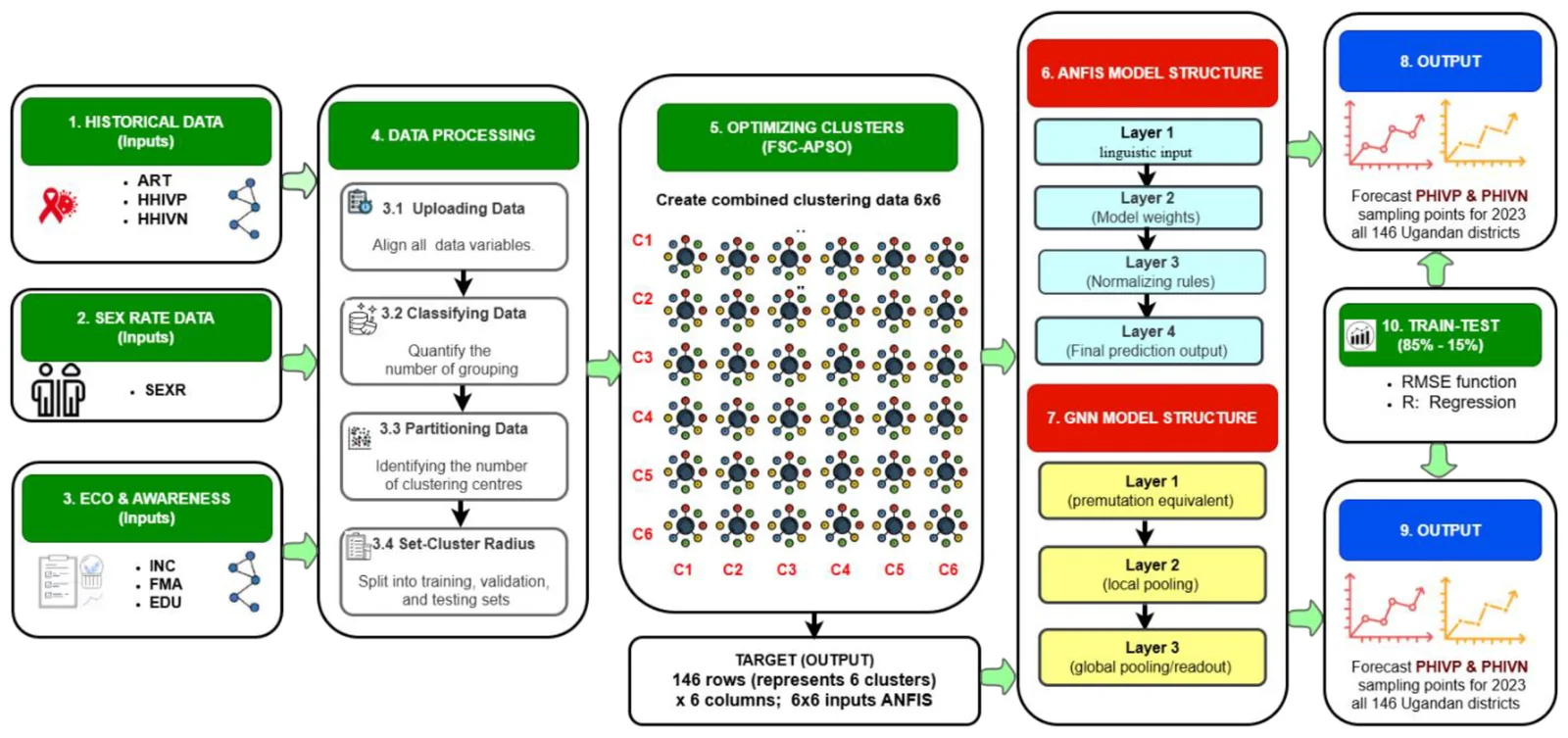
The proposed study’s framework for predicting and tracking PHIVP and PHIVN. The figure shows the flowchart of district-level 2023 HIV data selection and analysis by using the FSC–APSO–ANFIS (denoted as scenario I) technique. Also, the flowchart includes the improved ANFIS (scenario II) and GNN (scenario III) approaches. This flowchart describes how the simulation implemented datasets for the predictive tracking PHIVP and PHIVN system for all 146 Ugandan districts, facts on HIV/AIDS in Uganda 2023. Each model (PHIVP/PHIVN) has 6 variable inputs with 6 clusters (*C*_i_). AIDS = acquired immune deficiency syndrome, ANFIS = adaptive neuro-fuzzy inference system, APSO =accelerated particle swarm optimization, ART = antiretroviral therapy, EDU = sex education, FMA = family member awareness, FSC = fuzzy subtractive clustering, GNN = graph neural networks, HHIVN = historical HIV new incidence, HHIVP = historical HIV prevalence, HIV = human immunodeficiency virus, INC = individual income, PHIVN = new HIV incidence, PHIVP = HIV prevalence, RMSE = root mean square error, SEXR = sex rate.

The framework of this modeling study of the district-level 2023 HIV data for predicting PHIVP and tracking PHIVN is shown in Figure 1. It has been carried out using the general steps approach as follows: data collection and variable identification, data clustering and optimization, and data modeling and prediction. For data prediction, 3 AI methods have been adopted to track the prediction of PHIVP and PHIVN, which are GNNs,^[21]^ improved ANFIS,^[47]^ and the proposed FSC–APSO–ANFIS method.

**Figure 2.**
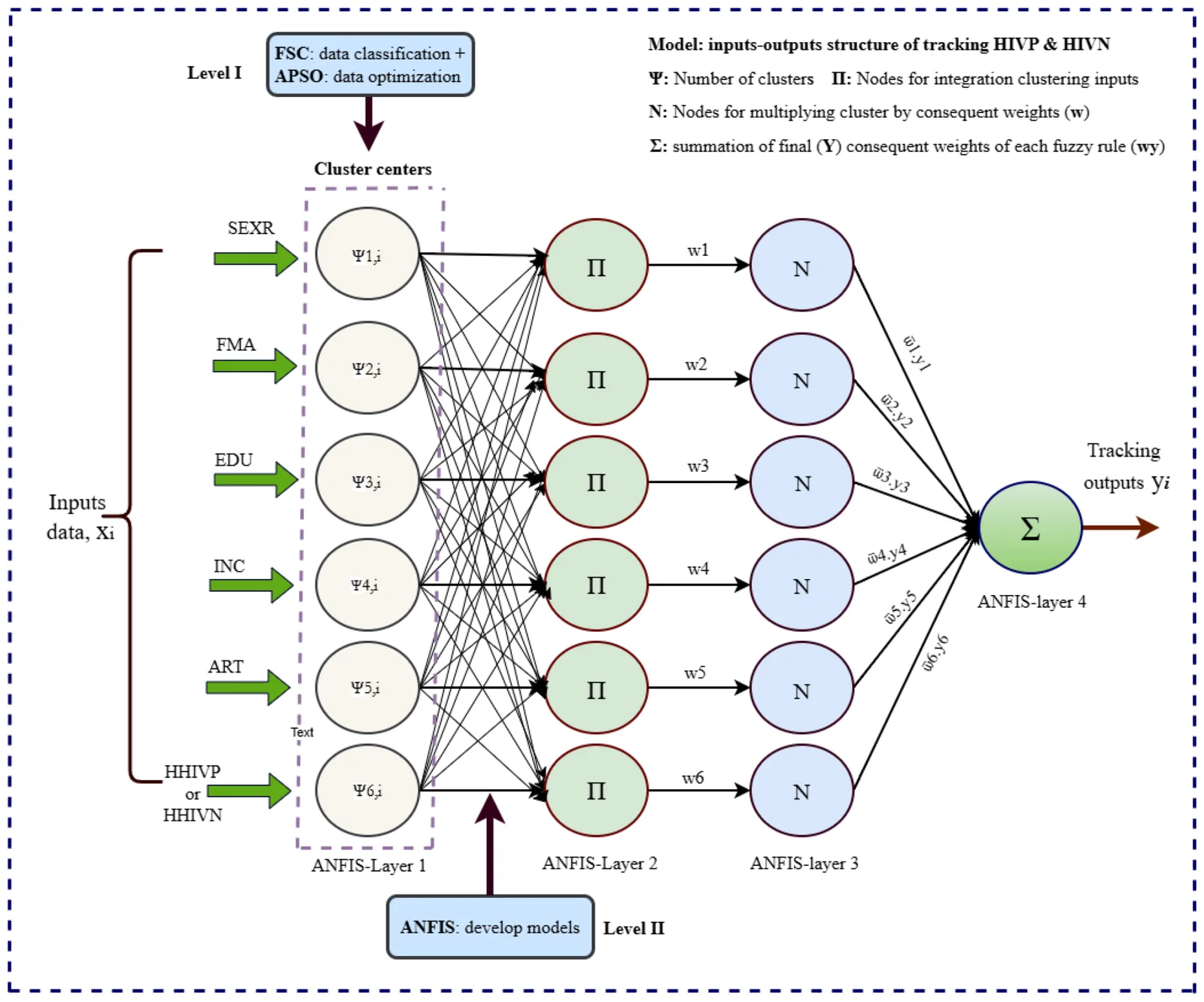
Inputs–outputs: HIV prevalence and new infections using improved ANFIS. The figure illustrates a 6-input**–**output diagram of the optimized 4-layer ANFIS structure with 2 intelligent levels. Level I employed the FSC–APSO algorithm to divide and group datasets into clusters (Ψ_*ij*_), while level II used ANFIS for building a predictive tracking model (*y*_*i*_). Layer 1 is a linguistic input (*x*_*i*_), layer 2 is a linear polynomial of input data with weights (*w*_*i*_), layer 3 is used to create fuzzy rules, and layer 4 is the final output for the predictive PHIVP and PHIVN models. FSC is fuzzy subtractive clustering, APSO is accelerated particle swarm optimization, ANFIS is an adaptive neuro-fuzzy inference system. ANFIS = adaptive neuro-fuzzy inference system, APSO =accelerated particle swarm optimization, ART = antiretroviral therapy, EDU = sex education, FMA = family member awareness, FSC = fuzzy subtractive clustering, HHIVN = historical HIV new incidence, HHIVP = historical HIV prevalence, HIV = human immunodeficiency virus, INC = individual income, PHIVN = new HIV incidence, PHIVP = HIV prevalence, SEXR = sex rate.

a) The models PHIVP and PHIVN have been evaluated using the GNNs technique. GNNs have become popular due to their capacity to process data in non-euclidean space in representations such as graphs with nodes and edges. The goal of the GNN is to learn from graph node representations so that predictions can be made.^[21]^ In this study, 85 % of the training data from the 6 datasets (Fig. 2) were used for projecting the tracking of PHIVP and PHIVN status, while the remaining 15% were used as testing data. Then, the GNN model training was performed through 300 iterations to be validated.
b) An ANFIS was then used to create a tracking model for PHIVP and PHIVN by utilizing the chosen optimum clusters as input data. ANFIS is a hybrid of an artificial neural network and fuzzy inference system (FIS), both of which are employed as expert systems owing to their interpretability and strong learning capabilities.^[48]^
c) The gathered data will be divided into 6 groups using FSC and then optimized by APSO; ANFIS uses the optimal clusters as input data to forecast the final PHIVP and PHIVN models.

#### 2.1.1. Data collection

All data used in this study were obtained from publicly available national HIV surveillance estimates and district-level data for 2023. According to a statistical analysis of 2023 data, there were approximately 38,132 PHIVN and a 5.1% PHIVP that year; see the following link: https://www.mnypa.org/docs/1723095739.pdf. Some data were generated randomly, and others were collected from the survey (questionnaires). There are 146 districts, which are further divided into 4 administrative regions^[49]^: the northern region (NR), eastern region (ER), western region (WR), and central region (CR), representing 41, 40, 38, and 27 districts, respectively (Appendix I, Supplemental Digital Content 3). Each of the 146 districts has historical data on HIV prevalence (HHIVP), historical data on HIV new incidence (HHIVN), and ART coverage, according to HIV 2023 estimates by the Ugandan AIDS commission. The historical data for HHIVP, HHIVN, and ART ranged from 3.8% to 13%, 0 to 38,132 cases, and 50% to 90%, respectively. The proportion of PLHIV covered by ART for CR, NR, WR, and ER was 84.1% (76.9%–>89.5%), 82.4% (48.3%–>89.8%), 80.6% (70.3%–>86.3%), and 80.5% (55.2%–>88.9%), respectively (https://www.mnypa.org/docs/1723095739.pdf). Additionally, we assumed that the FMA, EDU, and sex rate (SEXR) statistics ranged between 0 and 1, as did INC. For INC, we performed a survey on individual income in public and private organizations in some districts. We conducted a survey on INC in governmental and private entities across several districts. According to the poll, the average INC for those working in the public and private sectors varied from 375,000 (100) to 1,875,000 UGX (500 USD). The INC is divided into 6 categories: 50–100, 100–200, 200–300, 300–400, and 400–500 USD, in addition to unemployed individuals with 0.0 USD. After the INC survey, we created INC data for each district at random, ranging from 0.0 to 500 USD. We then used the random data and the gathered data (all 6 datasets) to estimate and predict PHIVP and PHIVN models.

#### 2.1.2. Data variables and identification

In this work, we targeted predicting PHIVP and PHIVN for people aged 15 to 49 years with HIV-associated factors. For the PLHIV category, to understand the gravity of HIV/AIDS, ART, FMA, and EDU are crucial factors.^[50,51]^ While EDU offers some protection against HIV, HIV prevalence and new infections are higher among older PLHIV who lack education, have no income, and have limited access to healthcare facilities.^[52]^ Preventive education and information are promoted using condoms and altering sexual behavior to reduce HIV infection rates, as well as treatment-based ART.^[53]^ The data collection process ensured completeness was identified for the key variables (HHIVP**/**HHIVN and ART). Additional influential factors were considered: SEXR, EDU, INC, and FMA. This study presents 2 predictive tracking models: PHIVN with influential determinants and PHIVP among PLHIV aged 15 to 49 years. To validate the predictive model, 85% of the 146 districts with the influential variables of 6 inputs (146 × 6) datasets were used as training data.

#### 2.1.3. Data optimization

This is used to determine the input 146 × 6 dataset, where 146 is the number of districts, and 6 is the number of input variables such as SEXR, FMA, EDU, INC, ART, HHIVP, and HHVIN. The inputs are combined with district numbers and can be used to estimate the spread of HIV and infections. These factors can directly impact the rates of PHIVP and PHIVN using neuro-fuzzy. To classify the dataset, FSC was adopted by searching for particular structures to estimate the number of clusters of PHIVP and PHIVN.^[48]^ The data that have been clustered by the FSC are optimized using APSO to select the best results. Whereas fish in schools and birds in flocks naturally create an atmosphere in which to find food, APSO draws inspiration from these behaviors.^[54]^

### 2.2. Data prediction-based FSC–APSO–ANFIS approach

**Figure 3.**
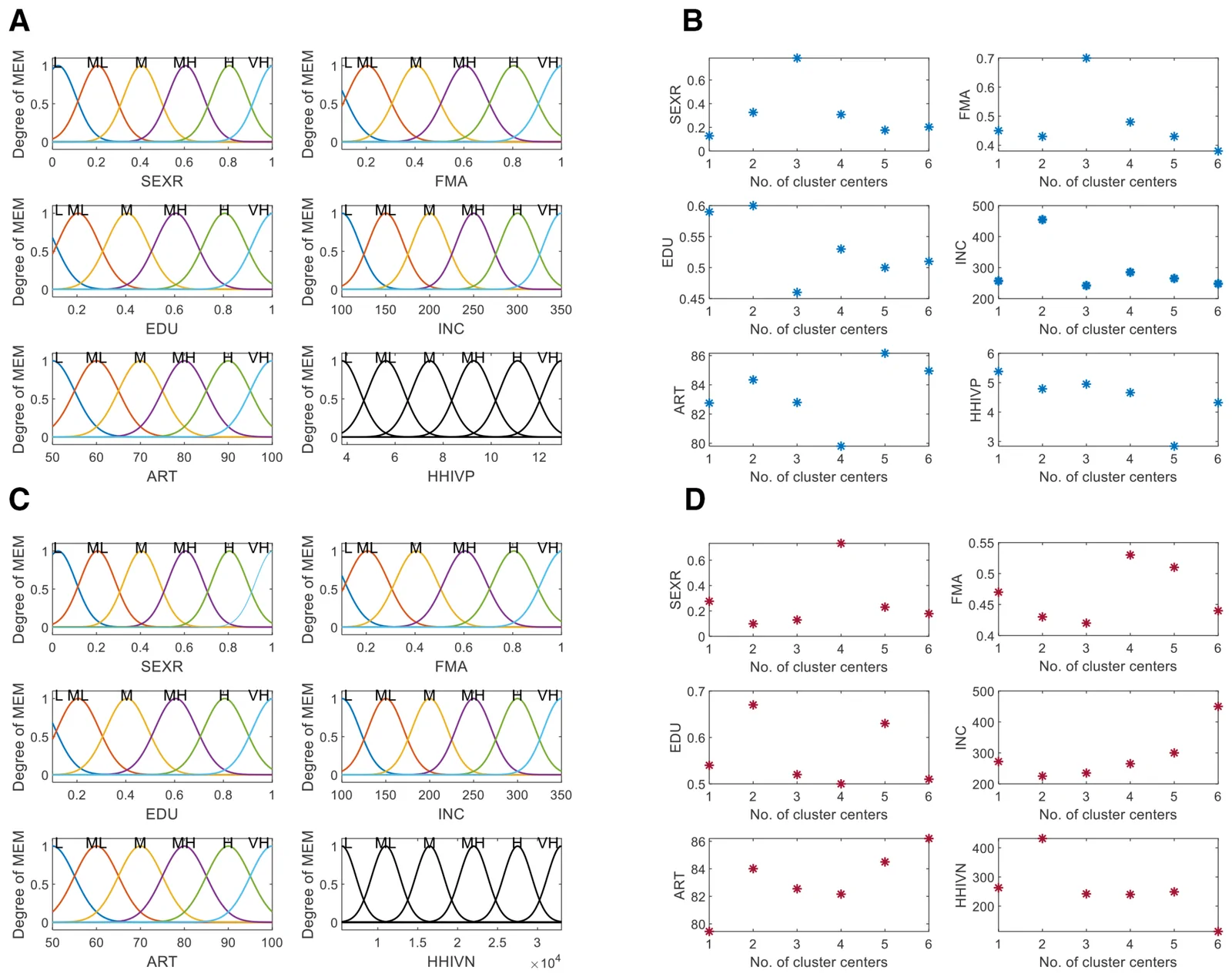
Data modeling: (A and B) fuzzy sets and memberships**–**optimal clustering centers of PHIVP, (C and D) fuzzy sets and memberships**–**optimal clustering centers of new PHIVN. The figure illustrates data modeling: (A and B) show 6 fuzzy sets and memberships and 6 optimal clustering centers of HIV prevalence “PHIVP”; (C and D) show 6 fuzzy sets and memberships and 6 optimal cluster centers of new incidence “PHIVN.” Fuzzy sets in (A–C) have different values ranging from low (L), medium low (ML), medium (M), medium high (MH), high (H), and very high (VH) with a degree of membership (MEM). The FSC–APSO algorithm adjusted the fuzzy sets into 6 cluster centers in B–D) to evaluate the PHIVP and PHIV. APSO =accelerated particle swarm optimization, ART = antiretroviral therapy, EDU = sex education, FMA = family member awareness, FSC = fuzzy subtractive clustering, HHIVN = historical HIV new incidence, HHIVP = historical HIV prevalence, HIV = human immunodeficiency virus, INC = individual income, PHIVN = new HIV incidence, PHIVP = HIV prevalence, SEXR = sex rate.

ANFIS-based optimal clustering, as AI, is used to develop a predictive tracking model-based dataset as inputs, as shown in Figure 2. The datasets are described as SEXR, ART, FMA, EDU, INC, and HHIVP or HHIVN. Approximately 15% of datasets out of 100% were used to test the prediction models of PHIVP and PHIVN using the optimal adaptive ANFIS technique. As shown in Figure 3A–C and B–D, the 2 models are composed of 6 inputs, each of which has 6 membership functions (MFs) integrated as fuzzy sets derived from the training data. Herein, a 2-level intelligent technique is involved in decision-making based on the selected optimal data featuring the FSC–APSO algorithm as the decision maker using the ANFIS technique. The implementation of a 2-level intelligent technique is detailed as follows.

#### 2.2.1. Level I: FSC–APSO algorithm

The FSC algorithm is one of the fuzzy applications that can be used to classify data and to estimate the number of data clusters by searching the collected data to select the best data center among data sampling points.^[48]^ In level I, let *x*_*l*_ = *x*_1_, *x*_2_,…, *x*_*n*_, where *x*_*i*_ represents the (Ψ_*ij*_) fuzzy sets of datasets, and each Ψ*ij* has 6 fuzzy rules, which can be expressed by Eq. (1). Each input has a range with a minimum and maximum value and is partitioned into 6 fuzzy sets. Each fuzzy rule is represented as a cluster center in the fuzzy sets shown in Figure 3A–C, which are readjusted by the FSC algorithm.


$$x_{l}=\left(\;\begin{array}{cccccc} \Psi_{1,1} & \Psi_{1,2} & \Psi_{1,3} & \Psi_{1,4}\; & \Psi_{1,5} & \Psi_{1,6}\\ \Psi_{2,1} & \Psi_{2,2} & \Psi_{2,3} & \Psi_{2,4} & \Psi_{2,5} & \Psi_{2,6}\\ \Psi_{3,1} & \Psi_{3,2} & \Psi_{3,3} & \Psi_{3,4} & \Psi_{3,5} & \Psi_{3,6}\\ \Psi_{4,1} & \Psi_{4,2} & \Psi_{4,3} & \Psi_{4,4} & \Psi_{4,5} & \Psi_{4,6}\\ \Psi_{5,1} & \Psi_{5,2} & \Psi_{5,3} & \Psi_{5,4} & \Psi_{5,5} & \Psi_{5,6}\\ \Psi_{6,1} & \Psi_{6,2} & \Psi_{6,3} & \Psi_{6,4} & \Psi_{6,5} & \Psi_{6,6} \end{array}\right)$$ (1)

The datasets of each fuzzy rule and its membership can be expressed as,


$$\begin{array}{c} fuzzy\text{-}rule\;\left(FR\right)=\left\{if\;x_{i}\;is\;\mu_{ik}\right\},\;then\;\left\{y_{i}=C_{k}\right\} \end{array}$$ (2)

where µ_*ik*_ represents the membership (MEM), *k* represents the number of clusters or MEM, *C*_*k*_ represents the cluster center, and *y*_*i*_ represents the final output data point. Each *x*_*i*_ in the data clustering has the potential to be assigned as a candidate cluster center using the FSC algorithm. The potential data point *x*_*i*_ is assigned as a representative of a candidate cluster center *C*_*k*_ from the data density as expressed in Eq. (3).^[54]^


$$\begin{array}{c} D_{l}=\underset{l=1}{\overset{6}{\sum }}\;\exp\left\{-\frac{{\left\|x_{l}-C_{k}\right\|}^{2}}{{\left(r_{a}/2\right)}^{2}}\right\} \end{array}$$ (3)

where *r*_*a*_ is the radius of the data cluster (0, 1). The radius *r*_*a*_ has a direct impact on optimizing the data to search for a better result. Thus, the APSO algorithm was used to adjust or optimize the clustering data center by varying the values of the cluster radius.

APSO is a version of PSO inspired by the birds’ flock and fish schools. It is used to simulate the foraging swarm of particles; each particle (*i*) is defined by its position (Ω_*ij*_) and velocity (*v*_*ij*_) at each iteration (τ) of the *j*th dimension. By initializing each data parameter and encoding it into particles, the datasets were partitioned, and each value was selected randomly within its assigned range. The particle vectors of the fuzzy MFs are allocated and assigned according to their particle velocity and position^[54]^;


$$\begin{array}{c} V_{ij}^{\left(\tau +1\right)}=V_{ij}^{\left(\tau \right)}+\alpha r_{ij}+\;\beta \left({gbest}_{j}^{\left(\tau \right)}-\Omega_{ij}^{\left(\tau \right)}\right) \end{array}$$ (4)

$$\begin{array}{c} \Omega_{ij}^{\left(\tau +1\right)}=\Omega_{ij}^{\left(\tau \right)}\left(1-\beta \right)+\beta \left({gbest}_{j}^{\left(\tau \right)}\right)+\alpha r_{ij} \end{array}$$ (5)

where gbest is the global best solution for all particles N_*p*_, *r*_*ij*_ is the random number (0, 1), and α and β are the acceleration parameters, typically ≤1. Each particle *i* generates membership grades of the inputs, and each input data has 6 membership degrees. The MF parameters are set using random values within the proper ranges, and the distributions after each input were evaluated to establish the boundaries. Consequently, the range is partitioned, and each cluster center point value is randomly selected within its respective partition. The FSC–APSO repositions the center points of the MFs to obtain and optimize the best candidate datasets shown in Figure 3B–D for use as ANFIS model inputs in level II to model PHIVP and PHIVN.

#### 2.2.2. Level II: adaptive neuro-fuzzy inference system: ANFIS technique

As illustrated in Figure 3B–D, the cluster centers selected from the datasets obtained by Level I serve as inputs for the 4 layers of the ANFIS technique, which is implemented as detailed in Appendix II, Supplemental Digital Content 4. Fuzzy rules (FR) are constructed using ANFIS as a 1st-order linear model, which can be represented by^[48]^;


$$\begin{array}{c} FR=\left\{\begin{array}{c} IF\{(x_{1}\;is\;\Psi_{i,1})\;a\;(x_{i2\;}is\;\Psi_{i,2})\;AND\;(x_{3\;}is\;\Psi_{i,3})\;AND\;(x_{4\;}is\;\Psi_{i,4})\\ AND\;(x_{5\;}is\;\Psi_{i,5})\;AND\;(x_{6\;}is\;\Psi_{i,6})\},\;THEN\\ y_{j}=\gamma_{i1}x_{i1}+\gamma_{i2}x_{i2}+\gamma_{i3}x_{i3}+\;\gamma_{i4}x_{i4}+\gamma_{i5}x_{i5}+\gamma_{i6}x_{i6}+\;\gamma_{i0} \end{array}\right\} \end{array}$$ (6)

where *y*_*j*_ represents the predictive tracking models of the PHIVP and PHIVN, and γ_*ij*_ is the premise parameter of the ANFIS model. The antecedent (Ψ_*ij*_) fuzzy set ranges over the linguistic terms {L: low value, ML: medium low, M: medium, MH: medium high, H: high, and VH: very high value}.

The ANFIS simulation was implemented using 2 data sets: training and testing. For training, the input data were used to create a matrix relationship and construct fuzzy MFs for each model. For each individual input data point, fuzzy sets (Ψ_*ij*_) consisted of 6 values: L, ML, M, MH, H, and VH. Each fuzzy model has 6 MFs for each input datum. The MFs and fuzzy sets corresponding to the fuzzy model of PHIVP and PHIVN were obtained from the training data and are illustrated in Figure 3A–C. The fuzzy sets and MFs were μΨ^FR^_*ij*_, where μ_SEXR_^6^_6*i*_ = {μ_L_, μ_ML_, μ_M_, μ_HM_, μ_H_, _VH_}, μ_FMA_^6^_6*i*_ = {μ_L_, μ_ML_, μ_M_, μ_HM_, μ_H_, _VH_}, μ_EDU_^6^_6*i*_ = {μ_L_, μ_ML_, μ_M_, μ_HM_, μ_H_, _VH_}, μ_INC_^6^_6*i*_ = {μ_L_, μ_ML_, μ_M_, μ_HM_, μ_H_, _VH_}, μ_ART_^6^_6*i*_ = {μ_L_, μ_ML_, μ_M_, μ_HM_, μ_H_, _VH_}, μ_{HHIVP or HHIVN}_^6^_6*i*_ = {μ_L_, μ_ML_, μ_M_, μ_HM_, μ_H_, _VH_} for the developed ANFIS models (PHIVP & PHIVN). The antecedents of the fuzzy rules are illustrated in Tables S1 and S2, Supplemental Digital Contents 1 and 2, and the consequences of the models obtained from the ANFIS training procedure are represented in Appendix III, Supplemental Digital Content 5. The fuzzy-rule explicit representation of PHIVP and PHIVN models is based on Eq. (6), as shown in Appendix IV, Supplemental Digital Content 4. Following 300 training data iterations, the final developed models were optimized and validated by data testing. When the RMSE reached the lowest value in both the training and testing data sets, which was found at 300 iterations, the models were determined to have met the requirements of well-based regression (*R*).

### 2.3. Simulation details of the FSC–APSO–ANFIS approach

#### 2.3.1. Data loading and definition

146 districts and data from 6 datasets (SEXR, ART, FMA, EDU, INC, and HHIVP or HHIVN) were uploaded as described (*x*_*i*_). About 85% of these datasets were utilized for training, and the remaining 15% were used for testing and data validation. The 6 fuzzy rules and their clusters are constructed using the parameters *k*: number of clusters, *C*: number of cluster centers, α and β: acceleration parameters, N_*p*_: number of particles (swarm), τ: number of iterations, and *r*_*a*_: radius of clusters.

#### 2.3.2. Parameter initialization for data clustering

Initialize the parameters *r*_*a*_, α, β, and create a swarm N_*p*_ matrix for *V*, Ω, and gbest to partition the input datasets into 6 clusters. Identify the number of clusters, “*k*,” and set the density of data. Then, calculate the 1st cluster center “*C*_1_” to the last cluster center “*C*_6_”and update FR in accordance with the MFs. Each MF’s cluster center is selected with the intention of dispersing the MFs over the entire range. Each “*C*” value is selected at random within its corresponding range after the range is partitioned. Set the cluster radii, create an objective function-based RMSE, calculate the “gbest,” and update the particle’s velocity “*V*” and its position “Ω.” The “*V*” values are generated at random for each iteration, and particle “Ω” is calculated for the optimal cluster centers using the cluster radius “*r*_*a*_.”

#### 2.3.3. Predictive data modeling

The clustering data for 6 input variables, each input having 146 data points, were simulated into training and testing data. In this regard, 22 districts (15%) of the dataset were used to validate the prediction model (PHIVP and PHIVN, Appendices IV, Supplemental Digital Content 6) that was created using the training data from 124 districts (85%). Based on 2 scenarios, the simulation’s performance is assessed using the objective function, RMSE.

##### 2.3.3.1. FSC–APSO parameter setting

The FSC–APSO algorithm’s settings are as follows: swarm = 50 particles, iterations = 300, cluster radius = 0.53; number of cluster centers = 6; and acceleration factors α and β were set at 0.48 and 0.65, respectively.

##### 2.3.3.2. GNN parameter setting

The GNN model’s parameters include: number of layers = nodes = 3, number of input features = 18, number of total parameters = 14 (number of weights = 12 and number of biases = 2), random initialization values ranged between –0.55 and +0.55, number of iterations = 300.

##### 2.3.3.3. ANFIS parameter setting

The ANFIS model’s parameters include neuron count = 93, fuzzy rule count = 6, linear parameters = 30, and nonlinear parameters = 42; number of iterations = 300. In all cases, these simulation parameters were applied.

##### 2.3.3.4. Predictive model summary

**Figure 4.**
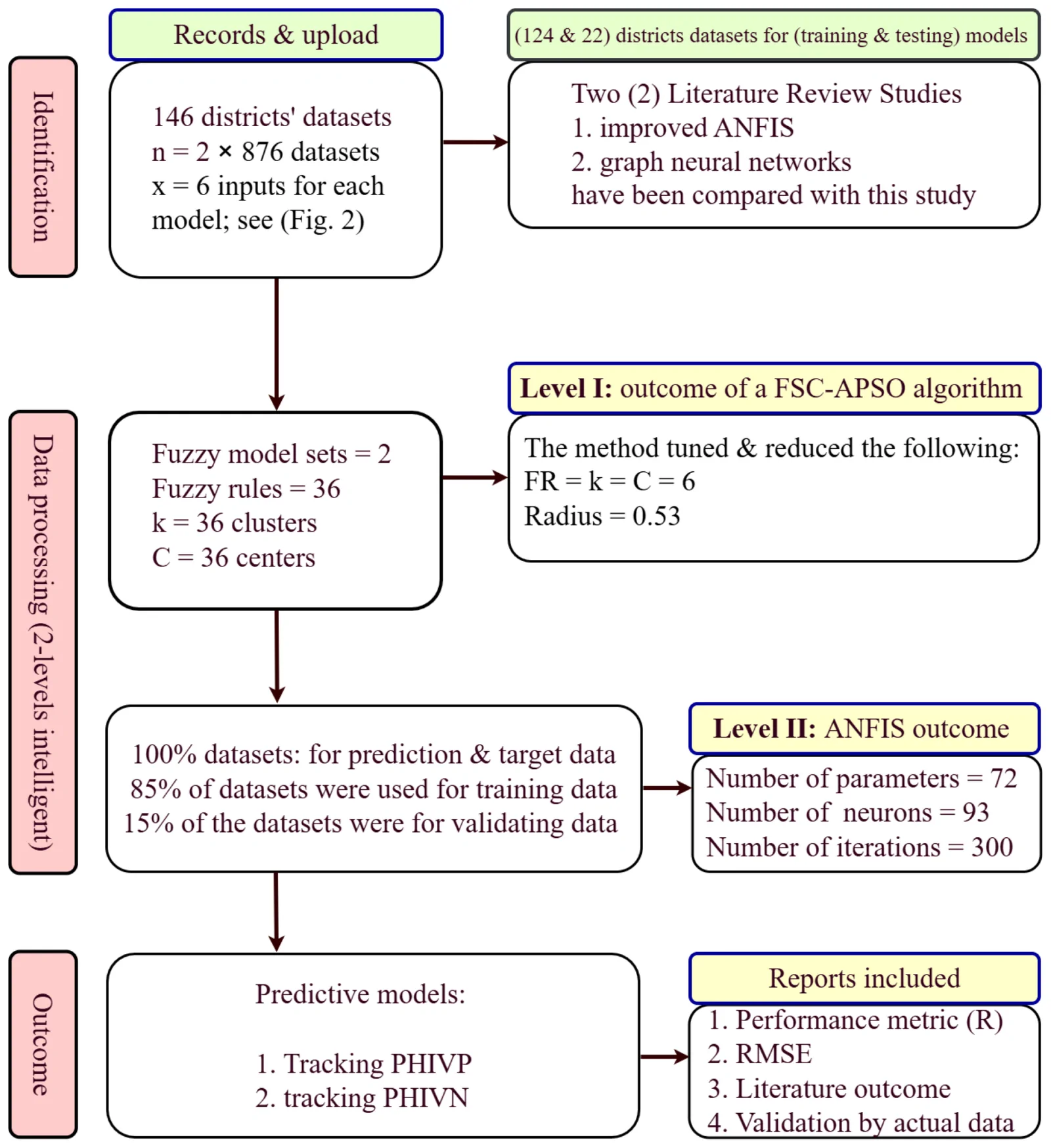
The proposed study’s flowchart numerical algorithm for tracking PHIVP and PHIVN. The figure shows the flowchart of the numerical algorithm for the implementation summary of datasets: data grouping/clustering, data optimization, and data prediction to assess the tracking model of HIV prevalence and new incidences of 146 Ugandan districts. ANFIS = adaptive neuro-fuzzy inference system, APSO =accelerated particle swarm optimization, FSC = fuzzy subtractive clustering, HIV = human immunodeficiency virus, PHIVN = new HIV incidence, PHIVP = HIV prevalence.

Figure 4 illustrates the 3 stages of the 2-level intelligent approach’s execution.

**Figure 5.**
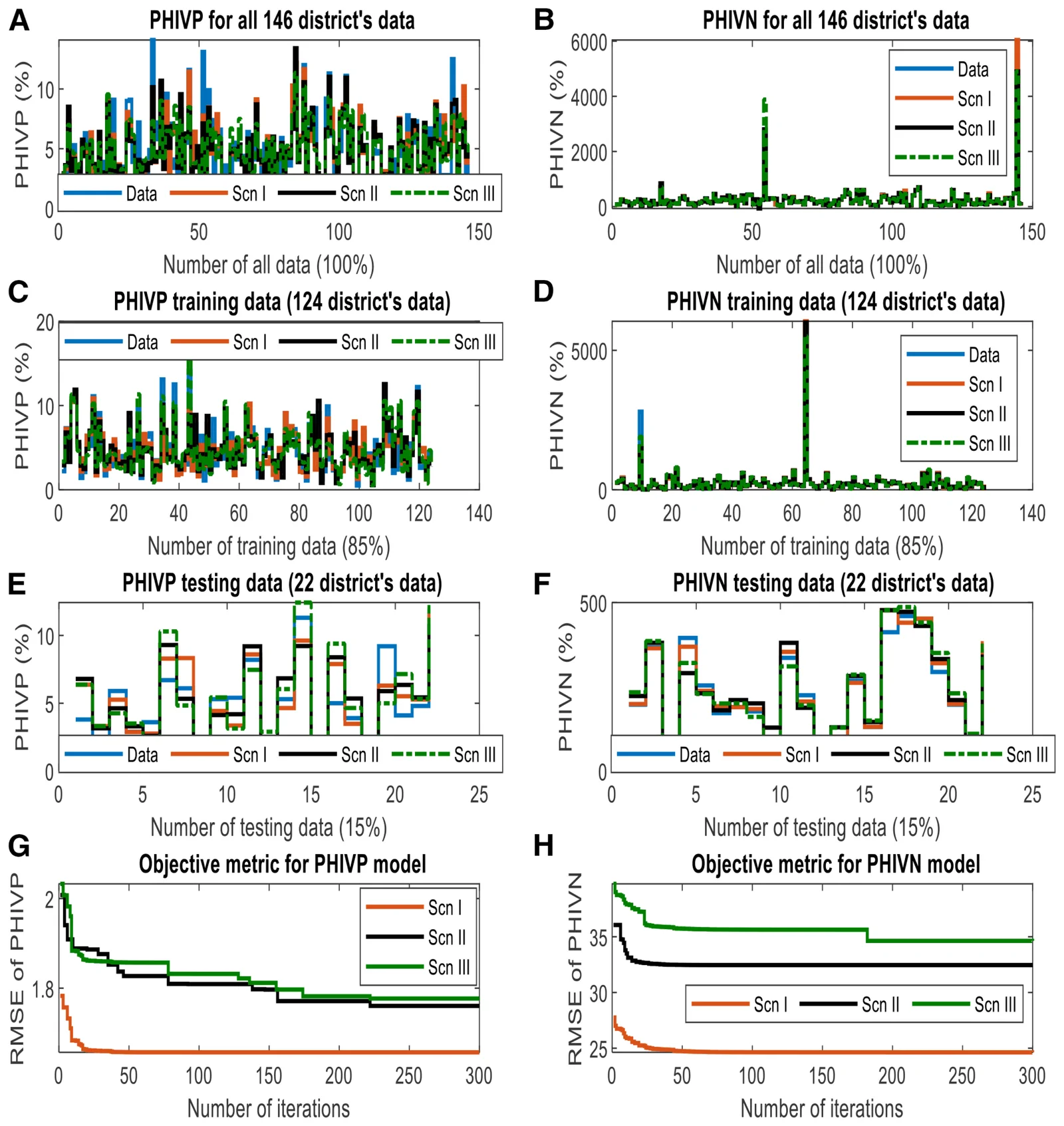
Prediction of PHIVP and tracking PHIVN: all data, testing data, and RMSE. The figure shows data modeling: (A and B) all districts predictive tracking evaluated by the regression (*R*),(C and D) 85% training predictive tracking,(E and F) 15% testing predictive tracking, and(G and H) objective metric as a RMSE for predictive tracking model of PHIVP and PHIVN, respectively. It compares the all data to 3 scenarios of the PHIVP and PHIVN model using objective performance-RMSE. The model trained and tested with the datasets with 300 iterations, where the models showed low errors. Scenario I gave lower RMSE values (RMSE = 1.657, 24.629) with *P* < .001 than scenario II (1.761, 32.457) and scenario III (1.776, 34.629) with *P* = .001 for HIV prevalence and new infections, respectively. *P* is the probability of observing a test statistic. Scenario I (FSC–APSO–ANFIS), scenario II (improved ANFIS), and scenario III (GNN). ANFIS = adaptive neuro-fuzzy inference system, APSO =accelerated particle swarm optimization, FSC = fuzzy subtractive clustering, GNN = graph neural networks, HIV = human immunodeficiency virus, PHIVN = new HIV incidence, PHIVP = HIV prevalence, RMSE = root mean square error, Scn I = scenario I), Scn II = scenario II, Scn III = scenario III. .

1. Determining the datasets, as shown in Figure 2, including inputs (6) for each data model. Two literature review studies, improved ANFIS and graph neural networks for evaluating HIV prevalence and new infections for PLHIV, have used data modeling.
2. The 2-level approach to data processing: level I and level II. Level I used the FSC technique to determine the number of clustering centers in Figure 3B–D after implementing the data grouping sets (fuzzy sets) indicated in Figure 3A–C. The cluster centers were then optimized by varying the cluster radii using the APSO approach. As illustrated in Figure 5A–F, level II uses 85% of the datasets for training data models and the remaining 15% for testing and validating data models.

**Figure 6.**
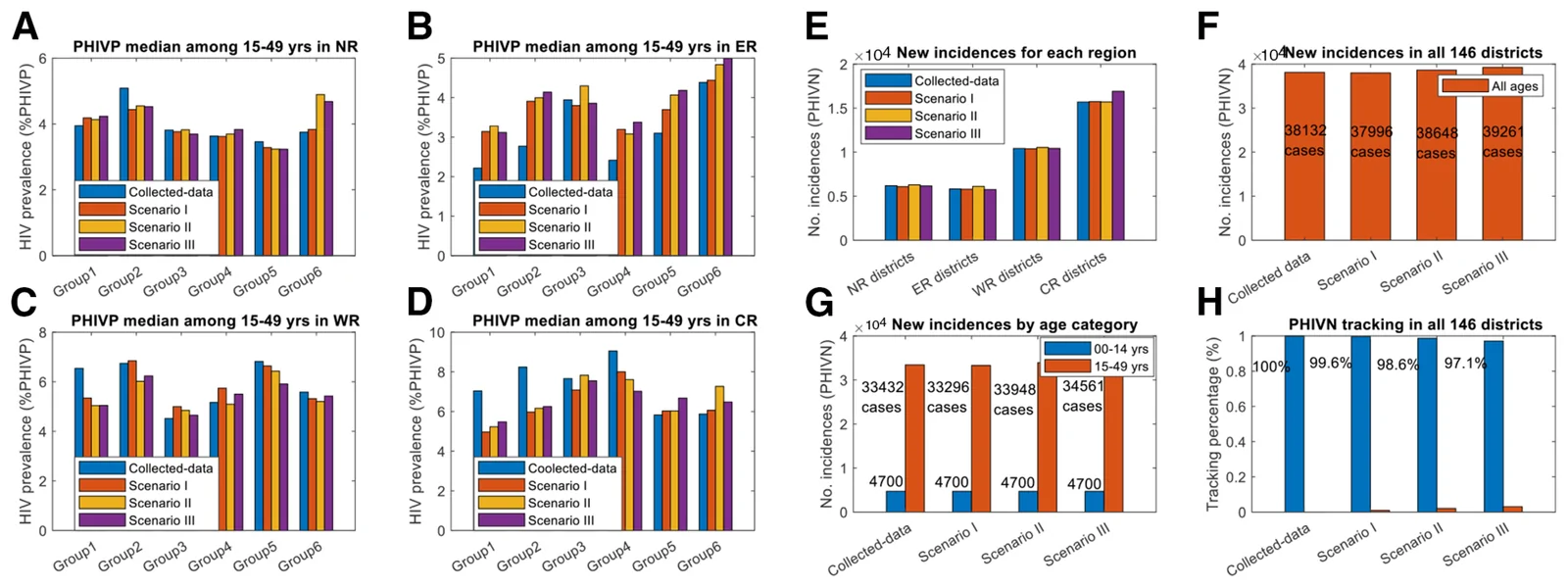
The median prediction: PHIVP and all PHIVN in 146 districts for individuals aged 15 to 49. The figure illustrates the prediction’s median: (A–D) HIV prevalence (PHIVP) among people aged 15 to 49 years in NR, ER, WR, and CR, respectively; and(E–H) median new HIV incidence by regional groups, all ages, 15 to 49 years, and tracking percentage, respectively. (A) PHIVP median for the NR of 41 districts that represents 6 groups. (B) PHIVP median for the ER of 40 districts that represents 6 groups. (C) PHIVP median for the WR of 38 districts that represents 6 groups. (D) PHIVP median for the CR of 27 districts that represents 6 groups. (E) Tracking PHIVN for each region (NR, ER, WR, and CR) by data collection versus scenario I, scenario II, and scenario III. (F) Tracking PHIVN for all regions by data collection versus scenario I versus scenario II versus scenario III. (G) Tracking PHIVN throughout Uganda (all 4 regions) among 15 to 49 years by data collection versus scenario I versus scenario II versus scenario III. (H) Percentage tracking PHIVN throughout Uganda (all 4 regions) for 15 to 49 years and all ages by data collection versus scenario I versus scenario II versus scenario III. CR = Uganda’s central region, ER = Uganda’s eastern region, HIV = human immunodeficiency virus, NR = Uganda’s northern region, PHIVN = new HIV incidence, PHIVP = HIV prevalence, WR = Uganda’s western region.

The applied models tracked PHIVP and PHIVN models using the parameters listed in Sections 2.3.3.1, 2.3.3.2, and 2.3.3.3. The accuracy and performance of the methodology showed its strength and dependability, as shown by the model regression (*R*), Figure 5G and H, and the performance metric (RMSE); see Table 3. Similar to the conclusions, the observational study revealed clear bitterness and consistency compared to previous studies in the literature review, with validation using actual data as depicted in Figure 6 and Table 3.

## 3. Results

### 3.1. Predictive model performance

As a result, the tracking models of the all-data, training data, and testing data closely matched the actual data, as shown in Figure 5A–F. Models’ performance is assessed using an objective metric, such as the RMSE displayed in Figure 5G and H, where the lowest RMSE indicates the highest model regression and better accuracy. Thus, RMSE can be expressed in Eq. (7),


$$\begin{array}{c} RMSE=\sqrt{\frac{{\left(collected\;data-predictive\;data\right)}^{2}}{N}} \end{array}$$ (7)

where N = 146 denotes the number of datasets, *y*_act_ = actual collected data, and *y*_pre_ = predicted data. Additionally, the coefficient of determination (*R*^2^) evaluates the overall model’s goodness, which is expressed in Eq. (8), *y*_mn_ = the data’s mean.


$$\begin{array}{c} R^{2}=1-\underset{i=1}{\overset{N}{\sum }}\;\frac{{\left(y_{act}-y_{pre}\right)}^{2}}{{\left(y_{act}-y_{mn}\right)}^{2}} \end{array}$$ (8)

Lower RMSE indicates higher predictive accuracy, while an *R*^2^ evaluates good agreement between actual and predicted data. As seen in Table 3, scenario I (FSC–APSO–ANFIS) performed better than comparable literature review studies when compared to the upgraded ANFIS (scenario II) and GNN (scenario III).

### 3.2. Comparative studies

We found that among people living with HIV aged 15 to 49 years across all 146 districts in Uganda, the estimated PHIVP individuals were 5.1%, and the PHIVN were estimated at 33,432 for the year 2023, based on actual data gathered from (https://www.mnypa.org/docs/1723095739.pdf) regarding HIV/AIDS facts. After accounting for influential variables among HIV-positive individuals, this study tracked PHIVN in 33,296 out of a total of 33,432 instances and estimated that the median PHIVP among the same set of people was 4.92%. In this study, a total of 146 × 6 district datasets (6 input data, each with 124 datasets) were included. The validation-based testing data comprised 22 district datasets (15%), of which 124 datasets (85%) were used to formulate the predictive and tracking models for PHIVP and PHIVN, respectively. As seen in Figure 5A and B, all datasets (100%) for PHIVP and PHIVN have been predicted in comparison with scenarios (I), (II), and obtained data. Figure 5C and D show that 85% of training datasets for PHIVP and PHIVN have been predicted, tested in Figure 5E and F, and evaluated with 300 iterations by RMSE in Figure 5G and H. Following training datasets, the estimated and tracked models PHIVP and PHIVN exhibit *P* values of <.001 (*P* < .001) for scenario I, *P* value = .001 (*P* = .001) for scenario II, and *P* value = .001 (*P* = .001) for scenario III, respectively. Using RMSE as an objective metric, the PHIVP–PHIVN were evaluated at 1.65 to 24.6, 1.76 to 32.4, and 1.77 to 34.6 by scenario I, scenario II, and scenario III, respectively. With a validation of 85.1% (*R* = 0.851) and an error of RMSE (1.6553) in Table 3, the proposal (scenario I) demonstrated its capacity to predict PHIVP as shown in Figure 5A, C, and E. Simultaneously, the same method has tracked the PHIVN extremely well, as demonstrated in Figure 5B, D, and F, with a testing performance of 98.7% (*R* = 0.987) in Table 3 and an error of RMSE in Figure 5H. In the district-level-based scenario, I showed a lower RMSE for PHIVP and PHIVN. For every district in Uganda, scenario I showed better tracking of PHIVP and PHIVN than scenario II and scenario III. In particular, the regressions for all data of PHIVP and PHIVN in scenario I were higher (0.823 and 0.987, respectively) than in scenario II (0.820 and 0.979) and then in scenario III (0.771 and 0.941). A simulation’s results demonstrated that the developed model-based scenario was a reliable and effective tool for predicting and tracking PHIVP and PHIVN. When comparing PHIVP and PHIVN with the obtained actual data, scenario I demonstrated its superiority over scenario II and scenario III.

### 3.3. Model analysis outcome

The optimal data for the PHIVP in this study are: the ART service was set to 83% (80%–>86%), the SEXR was 0.24 (0.18–0.66), the average INC was $242 ($225–$455), the EDU was 0.53 (0.5–0.67), the FMA was 0.47 (0.43–0.53), and the HHIVP or HHIVN were 4.8% (2.8%–5.4%) or 656 (106–38,132), respectively. The PHIVP’s median for each **group** was determined among 4 districts of Uganda (NR, ER, WR, and CR), see Appendix I, Supplemental Digital Content 3. Three scenarios (I, II, and III) in this observational study have been carried out and compared with the actual collected data, as illustrated in Figure 6A–D. The prediction model shows that most PHIVP in the NR, WR, and CR in Figure 6AD can estimate and track every district in those regions. Scenario I has consistently remained at or marginally above the actual PHIVP average in some groups (ER) shown in Figure 6B. However, when compared to the actual data collected, the results of scenarios I, II, and III in the ER regions were unable to track PHIVP in some districts. Scenario I’s ability to follow PHIVNs in regions with high HIV incidence further highlights its advantages. Scenarios II and III monitored 33,948 and 34,561 new incidents; however, they showed 516 and 1129 outbreaks, respectively. The scenarios (II and III) in Figure 6E were unable to track certain districts; scenario I tracked the number of PHIVN with a reduction of 136 cases (0.36%) for all ages and 15 to 49 years.

## 4. Discussion

### 4.1. Key findings

Using the Uganda AIDS commission’s 2023 datasets, 3 AI scenarios (models) were used in this work to forecast the PHIVP and PHIVN based on related parameters (eco, education, awareness, therapy, sex, and historical HIV). The performance of the models in scenario III (GNN)^[21]^ and scenario II (improved ANFIS),^[47]^ which utilized the data clustering matrix with 6 input datasets, showed reasonable findings, while scenario I indicated that it was more effective in applications requiring adaptability for tracking PHIVP and PHIVN. Despite the low values in PHIVN and PHIVP in the real-time environment, the results obtained by the scenarios (I, II, and III) showed a rise in new PHIVN and a high PHIVP% in the ER regions. In contrast to scenarios II and III (Tables 2 and 3, Fig. 6), the study model (scenario I) demonstrated improved tracking and higher accuracy in the NR, WR, and CR regions, despite an increase in PHIVP in some ER regions.

**Table 2:** PHIVP and PHIVN in Uganda’s 4 administrative regions (146 districts) for 2023.

NR = 41 districts Predictive PHIVP	Collected data Median: 3.95%	Scenario I Median: 3.85%	Scenario II^[47]^ Median: 4.05%	Scenario III^[21]^ Median: 4.03%
NR – districts group 1	3.9428	4.1829	4.1344	4.2318
NR – districts group 2	5.0857	4.4361	4.5485	4.5247
NR – districts group 3	3.8143	3.7648	3.8217	3.6936
NR – districts group 4	3.6285	3.6195	3.6951	3.8325
NR – districts group 5	3.4571	3.2832	3.2357	3.2330
NR – districts group 6	3.7500	3.8328	4.8918	4.6791

ER = 40 districts Predictive PHIVP	Collected data Median: 3.11%	Scenario I Median: 3.68%	Scenario II^[47]^ Median: 3.93%	Scenario III^[21]^ Median: 3.94%
ER – districts group 1	2.2143	3.1408	3.2783	3.1182
ER – districts group 2	2.7714	3.9076	3.9969	4.1380
ER – districts group 3	3.9428	3.7983	4.2968	3.8554
ER – districts group 4	2.4143	3.1946	3.0799	3.3772
ER – districts group 5	3.1000	3.6944	4.0663	4.1806
ER – districts s group 6	4.3833	4.4395	4.8344	4.9939

WR = 38 districts Predictive PHIVP	Collected data: Median: 5.89%	Scenario I Median: 5.81%	Scenario II^[47]^ Median: 5.44%	Scenario III^[21]^ Median: 5.46%
WR – districts group 1	6.5428	5.3431	5.0388	5.0466
WR – districts group 2	6.7428	6.8450	6.0179	6.2329
WR – districts group 3	4.5167	4.9980	4.8447	4.6494
WR – districts group 4	5.1667	5.7415	5.0952	5.5010
WR – districts group 5	6.8167	6.6391	6.4283	5.9103
WR–districts group 6	5.5833	5.3190	5.2036	5.4231

CR = 27 districts Predictive PHIVP	Collected data Median: 7.28%	Scenario I Median: 6.35%	Scenario II^[47]^ Median: 6.69%	Scenario III^[21]^ Median: 6.58%
CR – districts group 1	7.0400	4.9641	5.2314	5.4774
CR – districts group 2	8.2400	5.9704	6.1654	6.2495
CR – districts group 3	7.6600	7.0869	7.8283	7.5465
CR – districts group 4	9.0500	7.9990	7.6098	7.0191
CR – districts group 5	5.8250	6.0282	6.0277	6.6815
CR – districts group 6	5.8750	6.0627	7.2653	6.4814

Tracking PHIVN in districts	Collected data	Scenario I	Scenario II^[47]^	Scenario III^[21]^
NR	6192	6081	6289	6164
ER	5821	5790	6114	5757
WR	10,422	10,387	10,539	10,430
CR	15,697	15,738	15,707	16,911
Total of new incidence	38,132	37,996	38,649	39,261
PHIVN aged 0–14 yr	47,00	4700	4700	4700
PHIVN aged 15–49 yr	33,432	33,296	33,949^[47]^	34,561^[21]^

The data are presented as median for tracking PHIVN and estimating all PHIVN across Uganda regions. See Appendix I, Supplemental Digital Content 3. The overall findings of PHIVP and PHIVN: collected data = 5.1% and 33,432 incidence, scenario I = 4.9% and 33,296 incidence, scenario II = 5.03% and 33,949 incidence, and scenario III = 5.2% and 34,561 incidence respectively, among PLHIV aged 15-49 years.

Scenario I, scenario II, and scenario III represent (FSC–APSO–ANFIS), (improved ANFIS), and (GNN) respectively.

ANFIS = adaptive neuro-fuzzy inference system, APSO = accelerated particle swarm optimization, CR = Uganda’s central region, ER = Uganda’s eastern region, FSC = fuzzy subtractive clustering, GNN = graph neural networks, NR = Uganda’s northern region, PHIVN = new incidence of HIV, PHIVP = prevalence of HIV, PLHIV = people living with HIV, WR = Uganda’s western region.

**Table 3:** Performance metrics for PHIVP and PHIVN in Uganda’s 4 administrative regions (146 districts) for 2023.

Predictive model for PHIVP	Scenario I	Scenario II^[47]^	Scenario III^[21]^
RMSE: All data	1.6621	1.6674	1.8522
RMSE: Training data	1.6633	1.6502	1.8630
RMSE: Testing data	1.6553	1.7613	1.7903
Regression (*R*): All data	0.8230	0.8204	0.7729
Regression (*R*): Training data	0.8197	0.8199	0.7714
Regression (*R*): Testing data	0.8510	0.8232	0.7962

Predictive model for PHIVN	Scenario I	Scenario II^[47]^	Scenario III^[21]^
RMSE: All data	90.340	144.76	153.84
RMSE: Training data	97.478	156.48	166.14
RMSE: Testing data	24.608	32.615	34.629
Regression (*R*): All data	0.9875	0.9613	0.9409
Regression (*R*): Training data	0.9876	0.9611	0.9391
Regression (*R*): Testing data	0.9870	0.9794	0.9418

The data are presented as performance metric for tracking PHIVN and estimating all PHIVN across Uganda regions. Model error-based RMSE (root mean square error) and overall performance-based model regression (*R*): the higher the *R* (coefficient of correlation), the better the fitted model; the lower the RMSE, the higher the accuracy.

Scenario I, scenario II, and scenario III represent (FSC–APSO–ANFIS), (improved ANFIS), and (GNN) respectively.

ANFIS = adaptive neuro-fuzzy inference system, APSO = accelerated particle swarm optimization, FSC = fuzzy subtractive clustering, GNN = graph neural networks, PHIVN = new incidence of HIV, PHIVP = prevalence of HIV, RMSE = root mean square error.

### 4.2. Key findings argument

We compare the statistical data gathered from (https://www.mnypa.org/docs/1723095739.pdf) with the findings of PHIVP and PHIVN training using 3 adopted scenarios (I, II, and III)-based AI techniques. As reported, the actual median annual PHIVP among individuals (15–49) years in the NR ranged from lower to higher rates in Nabilatuk–Culu City, at approximately 3.95% (0.5%–10.5%), whereas the median PHIVP by scenario I, scenario II, and scenario III was 3.85%, 4.05%, and 4.03%, respectively, as given in Table 2. While the median PHIVP under scenario I, scenario II, and scenario III was 3.69%, 3.93%, and 3.94%, respectively, the actual median yearly of 2023 PHIVP in the ER in Pallisa–Soroti ranged from lower to higher, at approximately 3.14% (1.1%–12.5%). In contrast, the districts of WR and CR had median PHIVP of 5.81% and 6.35% under scenario I, 5.44% and 6.69% under scenario II, and 5.46% and 6.58% under scenario III, respectively. Meanwhile, the actual median PHIVP for people aged (15–49) in the districts of Bundibugyo–Fort Portal City and Buvuma–Kalangala in both regions varied from lower to higher, at approximately 5.89% (2.2%–14.1%) and 7.28% (4.0%–13.1%), respectively. In addition, scenarios II and III failed to track the decline in PHIVP in the ER, but they did lower the PHIVP in the other 3 regions. In general, the actual PHIVP is 5.1% (0.5%–14.1%). Scenarios (I, II, and III) estimated the overall PHIVP in 146 districts to be reduced by (4.92%, 5.03%, and 5.2%), respectively; therefore, scenarios I and II reduced the PHIVP by 3.53% and 1.37%, respectively, and failed in scenario III. Figure 6 illustrates the tracking of PHIVN of all ages, and it is observed that the proposed model (scenario I) in Figure 6E–G shows its ability to track every single district of each region and predict new incidences of PHIVN. While scenarios II and III were unable to track certain districts in WR and CR in Figure 6E. Scenario I reduced the number of PHIVNs by 136 cases (0.36%) for all ages and 15 to 49 years in Figure 6F and G after deducting 4700 PHIVNs for those aged 0 to 14 years, while II and III exceeded the actual data of new infections as shown in Table 2. The objective metric of RMSE was used to evaluate the overall performance index with 300 iterative processes for PHIVP and PHIVN models, as illustrated in Figure 5G and H, respectively, and depicted in Table 3. The analysis using scenario I demonstrated a high tracking of new incidence, with 99.6% for all ages and those aged 15 to 49; see Figure 6H. With outbreaks of 516 and 1129 cases, scenario II and scenario III tracked the new incidence with 98.6% and 97.1%, respectively. For PHIVP, both scenarios (I and II) performed well, estimating the PHIVP median, which matched the estimated statistical data by the UNAIDS Uganda HIV/AIDS commission.

### 4.3. Study strengths

The study-based FSC–APSO–ANFIS (scenario I) represented a potent computational tool and exhibited great strength and accuracy, which makes it superior to the regression and statistical models with a high regression value. Furthermore, the study is deemed adaptable and appropriate for any health outbreaks/epidemics. With a score of (*R* = 85.3%) for overall PHIVP prediction and (*R* = 98.7%) for tracking “PHIVN” across Uganda. To assess the robustness of our results, sensitivity analyses were performed by varying the cluster radius and number of particles in the FSC–APSO optimization process, as well as by altering the training-to-testing data ratio from 85:15 to 80:20. These variations did not materially affect the model’s predictive accuracy (changes in RMSE < 5%), suggesting that the findings are stable across reasonable parameter modifications. The study significantly allows authorized individuals to take action based on the tracking model, particularly in districts with a high rate of PHIVN that contribute to elevated PHIVP.

### 4.4. Study limitations

For huge data, the proposed technique requires a long simulation and computational time for training data, and when the number of iterations is small, the accuracy may be affected. While the sensitivity analyses were performed by altering the number of iterations from 300 to 200. The variations did materially affect the overall model performance (change in RMSE > 5, *R* = 0.812 for PHVIP and *R* = 0.954 for PHIVN); more iterations mean better accuracy. To improve accuracy and reduce performance time, strategies can be introduced. We recommend using a 5-layer ANFIS integrating with meta-heuristic algorithms.

### 4.5. Uganda’s progress based on 95–95–95 UNAIDS targets

ART therapy is essential for reducing PHIVP and avoiding PHIVN. In Uganda, as of 2023, estimates 92% of all PLHIV (15–49) years were aware of their status, 83% were on ART, and 79% were virally suppressed https://www.mnypa.org/docs/1723095739.pdf. As of right now, about 80.1% of PLHIV know their status, 96% have received ART, and 92% are virally suppressed; these developments underscore the significance of looking beyond the HIV cascade. The UNAIDS established the 95–95–95 targets, which call for 95% of all PLHIV to be diagnosed, 95% of those diagnosed with ART to be treated, and 95% of those treated to have viral suppression by 2030.^[55–57]^ With this progress, Uganda is on track to meet its 95–95–95 targets by 2030. Ugandan officials continued their efforts to reduce HIV prevalence, meet UNAIDS targets, and end the HIV/AIDS epidemic by 2030 despite the difficulties caused by the current US administration’s 24% USAID freeze (90 days) from SSA countries since January 2020, which has not yet resumed.

### 4.6. Generalizability

While this model was developed using Ugandan surveillance data, the methodological framework (FSC–APSO + ANFIS) is adaptable to other sub-Saharan African contexts or countries with similar HIV epidemiological profiles. However, generalization should be approached cautiously, as socioeconomic and health system factors may differ substantially.

## 5. Conclusions

This study offers and provides a powerful predictive tool for projecting HIV prevalence and new incidence in adults 15 + years old by using treatment, socio-data, and historical HIV data collected from https://www.mnypa.org/docs/1723095739.pdf in 2023. The study developed a general computational model with adequate agreement that has a robust tracking system (PHVIP–PHIVN: training dataset 85% with *R* = 0.832–0.987; PHVIP–PHIVN: validation dataset 15% with *R* = 0.851–0.987). Calibrating the RMSE to 1.6553 and 24.629 for PHIVP and PHIVN, respectively, supports the precision of predictive modeling for changes with an error <5%. After the data had been clustered, trained, and tested, the modeling study achieved high accuracy with PHIVP and PHIVN tracking of 99.6%. The modeling study reveals that it enhances data-driven decisions in addressing PLHIV in district regions where PHIVP and PHIVN are high. The findings of this study will be a baseline method for predicting the spread of HIV and tracking incidences in years to come. Also, it can help the authorities (lawmakers and health officials) in making decisions and policies in Uganda to meet UNAIDS targets and end the HIV/AIDS epidemic by 2030, such as implementing targeted prevention programs and increasing access to treatment for affected populations.

Supplemental Digital Contents “R4 AI Checklist, R4 TRIPOD-Checklist, Data collection of 146 districts IN Uganda” are available for this article.

## Acknowledgments

We are grateful to the Uganda AIDS Commission HIV for making PLHIV data publicly available on the website. Also, we are appreciative of everyone who provided survey data on individual income, awareness, and education to support our research. Our gratitude’s to Princess Nourah bint Abdulrahman University Researchers (PNURSP2026R764), for their support.

## Author contributions

**Conceptualization:** Elnazeer Ali Hamid Abdalla, Muna Elsadig, Amina Salhi.

**Data curation:** Val Hyginus Udoka Eze.

**Formal analysis:** Abuagla M. Dafalla.

**Funding acquisition:** Val Hyginus Udoka Eze.

**Investigation:** Elnazeer Ali Hamid Abdalla, Muna Elsadig.

**Methodology:** Elnazeer Ali Hamid Abdalla.

**Resources:** Muna Elsadig, Val Hyginus Udoka Eze, Amina Salhi, Abuagla M. Dafalla.

**Software:** Muna Elsadig.

**Validation:** Umi Omar, Val Hyginus Udoka Eze.

**Visualization:** Umi Omar.

**Writing – original draft:** Elnazeer Ali Hamid Abdalla, Muna Elsadig, Umi Omar, Val Hyginus Udoka Eze, Adil Mergani, Amina Salhi, Abuagla M. Dafalla, Yasreen Gasm Alkhalig, Sahla Mohammed Ahmed, Bienfait Mumbere Vahwere.

**Writing – review & editing:** Elnazeer Ali Hamid Abdalla, Muna Elsadig, Umi Omar, Val Hyginus Udoka Eze, Adil Mergani, Amina Salhi, Abuagla M. Dafalla, Yasreen Gasm Alkhalig, Sahla Mohammed Ahmed, Bienfait Mumbere Vahwere.


















