# Supplementary material for: Artificial neuro-fuzzy intelligence for dynamic tracking and prediction of HIV prevalence and new infections among individuals aged 15 to 49 years in Uganda: Computational national-level models
Source: Medicine (Baltimore). 2026 Sep 18;105(38):e50760. doi: 10.1097/MD.0000000000050760 (PMC13593017; doi:10.1097/MD.0000000000050760)
Supplement: Supplementary file 1 [file medi-105-e50760-s007.pdf]

Elnazeer Abdalla

revised manuscript 3

Quick Submit

Quick Submit

Western Campus

Document Details

Submission ID

trn:oid:::1:3612769924

Submission Date

Jul 17, 2026, 6:23 PM GMT+3

Download Date

Jul 17, 2026, 6:34 PM GMT+3

File Name

R3_Revised_Manucript_medicine.docx

File Size

116.1 KB

24 Pages

9,666 Words

52,653 Characters

*% detected as AI

AI detection includes the possibility of false positives. Although some text in this submission is likely AI generated, scores below the 20% threshold are not surfaced because they have a higher likelihood of false positives.

Caution: Review required.

It is essential to understand the limitations of AI detection before making decisions about a student's work. We encourage you to learn more about Turnitin's AI detection capabilities before using the tool.

Disclaimer
Our AI writing assessment is designed to help educators identify text that might be prepared by a generative AI tool. Our AI writing assessment may not always be accurate (i.e., our AI models may produce either false positive results or false negative results), so it should not be used as the sole basis for adverse actions against a student. It takes further scrutiny and human judgment in conjunction with an organization's application of its specific academic policies to determine whether any academic misconduct has occurred.

STROBE: Observational study

Artificial neuro-fuzzy intelligence for dynamic tracking and prediction of HIV prevalence and new infections among individuals aged 15 – 49 years in Uganda: Computational national-level models

Elnazeer Ali Hamid Abdalla, PhD[a], Muna Elsadig, PhD[b], Umi Omar, MD[c], Val Hyginus Udoka Eze, PhD[a,i,*], Adil Mergani, MD[d], Amina Salhi, PhD[b], Abuagla M. Dafalla, PhD[e], Yasreen Gasm Alkhalig, PhD[f], Sahla Mohammed Ahmed, MD[g], Bienfait Mumbere Vahwere, MD[h]

[a] Department of Electrical, Telecommunication and Computer Engineering, Kampala International University, P.O. Box 71, Ishaka, Uganda

[b] Department of Information Systems, College of Computer and Information Sciences, Princess Nourah bint Abdulrahman University, P.O. Box 84428, Riyadh 11671, Saudi Arabia

[c] CEO of Kampala International University Teaching Hospital and Research, P.O. Box 71, Ishaka, Uganda

[d] Department of Molecular Biology and Genetics, Kampala International University, P.O. Box 71, Ishaka, Uganda

[e] School of Allied Health Sciences, Kampala International University, P.O. Box 71, Ishaka, Uganda

[f] School of Architecture, Computing, and Engineering, University of East London, London E162RD, United Kingdom, UK

[g] Department of Physiology, Kampala International University, P.O. Box 71, Ishaka, Uganda

[h] Department of Surgery, Kampala International University, P.O. Box 71, Ishaka, Uganda

[i] Department of Electrical and Electronics Engineering, Mbarara University of Science and Technology, Kihumuro, Uganda

Corresponding author: udoka.eze@kiu.ac.ug* Department of Electrical and Electronics Engineering, Mbarara University of Science and Technology, Kihumuro, Uganda, (ORCID: 0000-0002-6764-1721)

Abbreviations: AI = Artificial intelligence; HIV = Human immunodeficiency virus; PLHIV = People living with HIV; PHIVP = HIV prevalence; PHIVN = New HIV incidence; ART = Antiretroviral therapy; SEXR = Sex rate; FMA = Family member awareness; EDU = Sex education; INC = Individual income; HHIVP = Historical HIV prevalence; HHIVN = Historical HIV new incidence; FSC = Fuzzy subtractive clustering; APSO =Accelerated Particle Swarm Optimization; ANFIS = daptive neuro-fuzzy inference system; NR = Uganda's northern region; ER = Uganda's eastern region; WR = Uganda's western region; CR = Uganda's central region; DHI = district health

information; EPP = Estimation and projection package; FIS = Fuzzy inference system; ANN = Artificial neural networks; RMSE =Root mean square error; iter =Iterations; MFs = Membership functions; FR = Fuzzy rules; GNN = Graph neural networks

Abstract

Background: Human immunodeficiency virus (HIV) infection remains a major global health burden, and Uganda continues to experience rising new HIV incidences, making prevention and management increasingly challenging. Accurate estimation of HIV prevalence (PHIVP) and tracking new HIV incidence (PHIVN) require robust computational models to effectively inform public health interventions.

Objectives: This study aims to develop a computational tracking model based on a two-level intelligent framework to dynamically monitor PHIVP and PHIVN in Uganda and to identify influential factors that contribute to HIV trends among individuals aged 15–49.

Methods: Six datasets from 146 Ugandan districts for 2023, sex rate, antiretroviral therapy coverage, awareness, education, personal income, and historical HIV indicators (prevalence and new incidence), were analysed. A two-level intelligent technique was employed: Level I, an upgraded clustering algorithm was used to identify optimal parameters influencing PHIVP and PHIVN; Level II, a neuro-fuzzy model trained these parameters to construct the computational tracking model. Data were partitioned into 85% for model training and 15% for testing and validation. Model accuracy was evaluated using regression performance, error metrics, and statistical significance.

Results: The model predicted a decline in PHIVP from 5.1% to 4.92%, and accurately tracked 37,996 PHIVN cases out of 38,132 actual incidences. The computational framework demonstrated strong predictive performance, yielding high regression values of R = 0.832–0.987 for PHIVP and PHIVN across three scenarios. Statistical analysis confirmed model reliability with $p < 0.001$ and $p = 0.001$.

Conclusion: The two-level intelligent approach exhibits strong potential for dynamically monitoring HIV prevalence and new incidence in Uganda. The six analysed datasets represent significant determinants of PHIVP reduction and PHIVN tracking among adults aged 15–49. These findings support the feasibility of computational modelling as a tool for enhancing HIV

surveillance, guiding prevention strategies, and strengthening evidence-based public health decision-making in Uganda.

Keywords: AI-based ANFIS; GNN; HIV prevalence; new HIV infection; people living with HIV

1. Introduction

According to the Uganda Ministry of Health's 2023 estimates, approximately 1,492,407 people are living with HIV (PLHIV—all ages), of which approximately 1.4 million are aged 15–49 years https://www.mnypa.org/docs/1723095739.pdf. HIV remains a major public health concern in Uganda due to persistent challenges such as sexual activity, economic inequality, and limited access to sexual health education [1]. HIV prevalence control efforts in Uganda are hampered by several challenges, including multiple sexual partners, poverty, which increases an individual's susceptibility to HIV, and knowledge gaps due to a lack of sex education [1]. Also, a lack of access to healthcare facilities for ART therapy, as well as a lack of affordability, knowledge, and education, are the main causes of the rising prevalence of HIV. However, over the past 15 years, Uganda has achieved tremendous progress in addressing the HIV decline using medical therapy-based antiretroviral therapy (ART).

Despite a decline in HIV prevalence (PHIVP) from 7.2% to 5.1% and new HIV incidence (PHIVN) from 69,000 to 33,000 incidences https://www.mnypa.org/docs/1723095739.pdf. The reduction in PHIVP and PHIVN has been largely attributed to ART, pre-exposure prophylaxis, awareness, and socioeconomic improvements [2-7]. While Uganda has implemented a number of preventative strategies to reduce HIV prevalence and new infections [1, 8], unsafe sex and multiple sexual behaviors continue to pose a serious risk to PLHIV (15–49 yrs). Numerous constraints pertaining to the data collection procedure and other systematic challenges limit the HIV statistics in Uganda that use numerical algorithms, hybrid prevalence estimates, and other regressions [9, 10].

1.1. Literature review: similar works

Artificial intelligence (AI) and machine learning (ML) offer promising applications in HIV response, including treatment planning, testing, early diagnosis, prediction, and monitoring of prevalence trends [11-15]. Considering the awareness and infection transmission knowledge, studies have demonstrated the potential of AI/ML in modeling HIV dynamics, optimizing ART dosing, and predicting infection patterns [12, 16-18]. For example, national-level modeling consortia have

developed tools to predict HIV prevalence, ART coverage, and stigma indices, whereas others have applied graph neural networks (GNNs) and empirical models for incidence prediction and prevalence estimation. District-level modeling systems have also been implemented to support diagnostic procedures and intervention planning [19-21]. Global studies further highlight variations in HIV prevalence by age, sex, and region, with the highest burden observed among PLHIV aged 25–54 years. Statistical models have been used to quantify gaps in ART coverage and vertical transmission prevention, whereas other models address virologic failure risks among adolescents by incorporating sociodemographic, behavioral, and economic factors. Analytical frameworks have additionally estimated prevalence and incidence based on ART status, mortality, and demographic profiles [18, 22-24]. Furthermore, multiple models have been formulated, aiming to estimate and predict new cases of HIV and its prevalence among PLHIV aged 15–49 years based on key populations [25]. In contrast, a number of systematic and analytical studies have been conducted to examine immunological and risk variables impacting HIV prevalence and new infections among PLHIV [26-29]. Study in [30] developed a prediction model to control the risk of virologic failure associated with HIV in teenagers. Treatment, sociodemographic, behavioral, psychological, and economic factors are among the variables that the model uses to control the exposure of anonymous and distinct HIV adolescents. Also, in analytical studies [31, 32], the rates of HIV prevalence and incidences of new HIV infections have been estimated using age and sex as well as on-ART therapy. Studies [33, 34] projected the PHIVP and PHIVN using different mathematical models. **Table 1** gives the summary of the PHIVP trend, including suggested methods, objectives, key findings, and a call to action.

Although the statistical and deterministic compartmental models often fail to capture the non-linear data, but, stratified cluster sampling model-based Spectrum are two more robust techniques that have been employed in Uganda's population-based HIV impact assessment [8, 18, 35-38]. Their limitations have become increasingly prominent when processing PHIVP and PHIVN load data modeling. The projection of PHIVP and PHIVN data contains "seasonal (peak and valley) data clustering" driven by associated HIV factors. However, the status awareness and ART factors are used to project the statistical models for PHIVP and PHIVN in the district/population level-based survey utilizing stratified cluster, DHI software, EPP/spectrum, and *prevR* method [38-40]. These are restricted to estimating small areas, and it found that the methods are unreliable and imprecise due to the uncertainty surrounding the validity of HIV estimates in specific districts or regions.

Despite using GNN (as an AI) to reduce HIV prevalence, it does not include validation datasets to support its conclusions [21]. To obtain accurate and trustworthy HIV statistics in Uganda, these limitations underscore the effectiveness of continuous efforts to improve data quality assurance and enhance data training for workers. Nevertheless, accurate prediction and tracking models are essential for informing public health policies and optimizing interventions [18, 41, 42]. The application of AI-based ANFIS models can capture nonlinear data effectively; however, there is no AI-based ANFIS study in Uganda to address HIV prevalence and new incidence, despite its success in epidemiology and healthcare. This study aimed to develop novel AI-based prediction model to track and estimate HIV prevalence (PHIVP) and new incidence (PHIVN) in Uganda, with a focus on supporting evidence-based strategies for sustaining control measures and enhancing national response efforts.

This research work intends to fill these research gaps by developing a dynamic ANFIS-based upgraded clustering algorithm for tracking PHIVN and PHIVN in 146 Ugandan districts based on 2023 historical supported data. This research, therefore, has the intention of making a scientific contribution with a significant impact on HIV prevalence and new infections. The major contributions of this study are summarized as follows:

- Development of a novel FSC–APSO–ANFIS framework that integrates the evolutionary symbolic learning capability of upgraded clustering (FSC–APSO) with the adaptive nonlinear inference strength of ANFIS, addressing a critical gap in PHIVP & PHIVN prediction studies.

- Comparative benchmarking against advanced AI, including FSC-ANFIS and FSC-APSO-ANFIS, with existing 2023 Uganda data using performance metrics (RMSE, regression, and model reliability).

- Provision of a reliable technique approach offering dynamic tracking of PLHIV to improve and reduce the spread of HIV and its incidences in a comparison with GNN and enhanced ANFIS models found in refs. [21] and [43] respectively, as well as the 2023 statistical data obtained from

This projecting model's study contributes to the body of knowledge by providing associated factors that can assist in identifying the lowest and highest HIV incidences and projecting the

overall prevalence percentage in Uganda. The findings of tracking HIV are significant for allocating resources by optimizing ART, increasing awareness, and maximizing educational tools to enable decision-makers to create an immediate action plan.

1.2. PLHIV action plan in Uganda

Young people, men, parents, leaders, and the community were urged by the Ugandan government and the Uganda AIDS Commission (https://www.mnypa.org/docs/1723095739.pdf) to take immediate action to reduce the spread of HIV infection and lower the number of PLHIV by implementing the following sustainable measures:

- 1.2.1. Using condoms when having sex
- 1.2.2. Education, awareness-raising, and providing free ART to people living with HIV.
- 1.2.3. Newly born must separate from the mother separation and decline breastfeeding
- 1.2.4. Safe male circumcision provides significant protection against HIV infections
- 1.2.5. Stopping HIV oral lesions, stopping defilement, and stopping domestic violence.

The increase of family awareness (FMA), educational level (EDU), and individual income rate (INC) has a positive impact on the decline of PLHIV. Moreover, the treatment via ART is a must to prevent HIV transmission from mother to child. To track overall HIV prevalence (PHIVP) and identify new incidence (PHIVN), the proposed model would be carried out in three phases. These phases include data collection and identification, data optimization, and data prediction.

2. Methods

2.1. Overview

The framework of this modeling study of the district-level 2023 HIV data for predicting PHIVP and tracking PHIVN is shown in **Figure 1.** It has been carried out using the general steps approach as follows: (1) data collection and variable identification, (2) data clustering and optimization, and (3) data modeling prediction. For data prediction, three AI methods have been adopted to track the prediction of PHIVP and PHIVN, which are graph neural networks (GNNs) [21], improved adaptive neuro-fuzzy inference systems (ANFIS) [43], and the proposed FSC-APSO-ANFIS method.

a) The models PHIVP and PHIVN have been evaluated using Graph neural networks (GNNs) technique. GNNs have become popular due to their capacity to process data in non-Euclidean space in representations such as graphs with nodes and edges. The goal of the GNN is to learn from graph node representations so that predictions can be made [21]. In this study, 85 % of the

training data from the 6 datasets (see **Figure 2**) were used for projecting the tracking of PHIVP and PHIVN status, while the remaining (15%) used testing data. Then, the GNN model training is performed through 300 iterations to be validated.

b) An ANFIS was then used to create a tracking model for HIV prevalence (PHIVP) and new infections (PHIVN) by utilizing the chosen optimum clusters as input data. ANFIS is a hybrid of an artificial neural network (ANN) and fuzzy inference system (FIS), both of which are employed as expert systems owing to their interpretability and strong learning capabilities [44].

c) The gathered data will be divided into six groups using FSC and then optimized by APSO; ANFIS uses the optimal clusters as input data to forecast the final PHIVP and PHIVN models.

2.1.1. Data collection

All data used in this study were obtained from publicly available national HIV surveillance estimates and district-level data for 2023. According to a statistical analysis of the 2023 data, there were approximately 38,132 new infections (incidences) and 5.1% of HIV prevalence in that year, see the following link https://www.mnypa.org/docs/1723095739.pdf. Some data have generated randomly and from the survey (questionnaires). There are 146 districts, which are further divided into four administrative regions [45]: the northern region (NR), eastern region (ER), western region (WR), and central region (CR), representing 41, 40, 38, and 27 districts, respectively, (**Appendix I**). Each of the 146 districts has historical data on HIV prevalence (HHIVP), new incidence (HHIVN), and ART coverage, according to HIV 2023 estimates by the Ugandan AIDS Commission. The historical data of HHIVP, HHIVN, and ART ranged from 3.8 to 13%, 0 to 38,132 cases, and 50 to 90%, respectively. The proportion of PLHIV covered by ART for CR, NR, WR, and ER was 84.1% (76.9->89.5%), 82.4% (48.3->89.8%), 80.6% (70.3->86.3%), and 80.5% (55.2->88.9%), respectively, (https://www.mnypa.org/docs/1723095739.pdf). Additionally, we assumed that the FMA, EDU, and SEXR statistics ranged between 0 and 1, as well as the INC. For INC, we performed a survey on individual income in public and private organizations in some districts. We conducted a survey on INC in governmental and private entities across several districts. According to the poll, the average INC for those working in the public and private sectors varied from 375,000 UGX (100 USD) to 1,875,000 UGX (500 USD). The INC is divided into six categories: (50–100) USD, (100–200) USD, (200–300) USD, (300–400) USD, and (400–500) USD, in addition to unemployed individuals with 0.0 USD. After the INC survey, we created INC

data for each district at random, ranging from 0.0 USD to 500 USD. We then used the random data and the gathered data (all 6 datasets) to estimate and predict PHIVP and PHIVN models.

2.1.2. Data variables and identification

In this work, we targeted predicting PHIVP and PHIVN for people aged 15 – 49 years with HIV-associated factors. For PLHIV category, to understand the gravity of HIV/AIDS, ART, FMA, and EDU are crucial factors [46, 47]. While EDU offers some protection against HIV, HIV prevalence and new infections are higher among older PLHIV who lack education, have no income, and have limited access to healthcare facilities [48]. Preventive education and information are promoted using condoms and altering sexual behavior to reduce HIV infection rates, as well as the treatment-based ART [49]. The data collection process ensured completeness was identified for the key variables (HHIVP/HHIVN and ART). Additional influential factors were considered: sex rate (SEXR), EDU, INC, and FMA. This study presents two predictive tracking models: new HIV infections (PHIVN) with influential determinants and HIV prevalence (PHIVP) among PLHIV aged 15-49 years. To validate the predictive model, 85% of the 146 districts with the influential variables of 6 input (146x6) datasets were used as training data.

2.1.3. Data optimization

This is used to determine the input 146×6 dataset, where 146 is the number of districts, and 6 is the factor of input variables such as SEXR, FMA, EDU, INC, ART, historical HIV prevalence (HHIVP), and new infections (HHVIN). The inputs are combined with district numbers can be used to estimate the spread of HIV and infection. These factors can directly impact their rates of PHIVP and PHIVN using neuro-fuzzy. To classify the dataset, fuzzy subtractive clustering (FSC) was adopted by searching using particular structures to estimate the number of clusters of PHIVP and PHIVN [44]. The clustering data that have been clustered by the FSC are optimized using accelerated particle swarm optimization (APSO) to select the best results. Whereas fish in schools and birds in flocks naturally create an atmosphere in which to find food, APSO draws inspiration from these behaviors [50].

2.2. Data prediction-based FSC-APSO-ANFIS

2.2.1. FSC-APSO-ANFIS Overview

Figure 3 illustrates the three stages of the two-level intelligent approach's execution.

1. Determining the datasets, as shown in Fig. 2, including inputs (6) for each data model. Two literature review studies—improved ANFIS and graph neural networks for evaluating HIV prevalence and new infections for PLHIV—have used the data modeling.
2. The two-level approach to data processing, level I and level II. Level I used the FSC technique to determine the number of clustering centers in Fig. 4(b–d) after implementing the data grouping sets (fuzzy sets) indicated in Fig. 4(a–c). The cluster centers were then optimized by varying the cluster radiuses using the APSO approach. As illustrated in Fig. 5(a–b–c–d–e–f), level II uses 85% of the datasets for training data models and the remaining 15% for testing and validating data models.
3. The applied models tracked PHIVP and PHIVN models using the parameters listed in section 2.4.4.4. The accuracy and performance of the methodology showed its strength and dependability, as shown by the model regression (R^2), **Figure 5(g-h),** and the performance metric (RMSE); see Table 3. Similar to the conclusions, the observational study revealed clear bitterness and consistency compared to the previous studies in the literature review.

ANFIS-based optimal clustering as AI used to develop a predictive tracking model-based dataset as inputs shown in **Figure 2**. The datasets are described as SEXR, ART, FMA, EDU, INC, and HHIVP or HHIVN. Approximately 15% of datasets out of 100% were used to test the prediction models of PHIVP and PHIVN using the optimal adaptive ANFIS technique. As shown in **Figure 4(a)–(c) & (b)–(d)**, the two models are composed of six inputs, each of which has six membership functions (MFs) integrated as fuzzy sets derived from the training data. Herein, a two-level intelligent technique is involved in decision making based on the selected optimal data featuring the FSC-APSO algorithm to be a decision maker using the ANFIS technique. The implementation of a two-level intelligent technique is detailed as follows:

2.2.1. Level I: FSC-APSO algorithm

The fuzzy subtractive clustering (FSC) algorithm is one of the fuzzy applications that can be used to classify data and to estimate the number of data clusters by searching the collected data to select the best data center among data sampling points [44]. In level I, let $x_l = x_1, x_2, \ldots, x_n$, where x_i represents the (Ψ_{ij}) fuzzy sets of datasets, and each Ψij has six fuzzy rules, which can be expressed by Eq. (1). Each input has a range with a minimum and maximum value and is partitioned into 6

fuzzy sets. Each fuzzy rule is represented as a cluster center in the fuzzy sets shown in **Figure 4(a)–(c),** which are readjusted by the FSC algorithm.

$$x_l = \begin{pmatrix} \Psi_{1,1} & \Psi_{1,2} & \Psi_{1,3} & \Psi_{1,4} & \Psi_{1,5} & \Psi_{1,6} \\ \Psi_{2,1} & \Psi_{2,2} & \Psi_{2,3} & \Psi_{2,4} & \Psi_{2,5} & \Psi_{2,6} \\ \Psi_{3,1} & \Psi_{3,2} & \Psi_{3,3} & \Psi_{3,4} & \Psi_{3,5} & \Psi_{3,6} \\ \Psi_{4,1} & \Psi_{4,2} & \Psi_{4,3} & \Psi_{4,4} & \Psi_{4,5} & \Psi_{4,6} \\ \Psi_{5,1} & \Psi_{5,2} & \Psi_{5,3} & \Psi_{5,4} & \Psi_{5,5} & \Psi_{5,6} \\ \Psi_{6,1} & \Psi_{6,2} & \Psi_{6,3} & \Psi_{6,4} & \Psi_{6,5} & \Psi_{6,6} \end{pmatrix} \quad (1)$$

The datasets of each fuzzy rule and its membership can be expressed as,

$$fuzzy - rule\ (FR) = \{if\ x_i\ is\ \mu_{ik}\}, then\ \{y_i = C_k\} \quad (2)$$

where μ_{ik} represents the membership (MEM), k represents the number of clusters or MEM, C_k represents the cluster center, and y_i represents the final output data point. Each x_i in the data clustering has the potential to be assigned as a candidate cluster center using the FSC algorithm. The potential data xi assigns as a representative of a candidate data center C_k from the data density as expressed in Eq. (3) [50].

$$D_l = \sum_{l=1}^{6} exp\left\{-\frac{\|x_l - C_k\|^2}{(r_a/2)^2}\right\} \quad (3)$$

where r_a is the radius of the data cluster (0, 1). The radius r_a has a direct impact on optimizing the data to search for a better result. Thus, the APSO algorithm was used to adjust or optimize the clustering data center by varying the values of the cluster radius.

Accelerated particle swarm optimization (APSO) is a version of PSO inspired by the birds' flock and fish schools. It is used to simulate the foraging swarm of particles, each particle (*i*) is defined by its position (Ω_{ij}) and velocity (v_{ij}) at each iteration (τ) of jth dimension. By initializing each data parameter and encoding it into particles, the datasets were partitioned, and each value was selected randomly within its assigned range. The particle vectors of the fuzzy membership functions (MFs) are allocated and assigned according to their particle velocity and position [50];

$$V_{ij}^{(\tau+1)} = V_{ij}^{(\tau)} + \alpha r_{ij} + \beta\left(gbest_j^{(\tau)} - \Omega_{ij}^{(\tau)}\right) \quad (4)$$

$$\Omega_{ij}^{(\tau+1)} = \Omega_{ij}^{(\tau)}(1-\beta) + \beta\left(gbest_j^{(\tau)}\right) + \alpha r_{ij} \quad (5)$$

where gbest is the global best solution for all particles Np, r_{ij} is the random number (0, 1), and α and β are the acceleration parameters, typically ≤ 1. Each particle *i* generates membership grades of the inputs, and each input data has 6 membership degrees. The MF parameters of the MFs are set using random values within the proper ranges, and the distributions after each input were

evaluated to establish the boundaries. Consequently, the range is partitioned, and each cluster center point value is randomly selected within its respective partition. The FSC-APSO repositions the center points of the MFs to obtain and optimize the best candidate datasets shown in **Figure 4(b)–(d)** for use as ANFIS model inputs in level II to model PHIVP and PHIVN.

2.2.2. Level II: Adaptive neuro-fuzzy inference system: ANFIS technique

As illustrated in **Figure 4(b)–(d)**, the cluster centers selected from the datasets obtained by Level I serve as inputs for the four layers of the ANFIS technique, which is implemented as detailed in **Appendix II**. Fuzzy rules (FR) are constructed using ANFIS as a first-order linear model, which can be represented by [44];

$$FR = \begin{Bmatrix} IF\{(x_1\ is\ \Psi_{i,1})\ a\ (x_{i2}\ is\ \Psi_{i,2})\ AND\ (x_3\ is\ \Psi_{i,3})\ AND\ (x_4\ is\ \Psi_{i,4}) \\ AND\ (x_5\ is\ \Psi_{i,5})\ AND\ (x_6\ is\ \Psi_{i,6})\}, THEN \\ \\ y_j\ = \gamma_{i1}x_{i1} + \gamma_{i2}x_{i2} + \gamma_{i3}x_{i3} + \gamma_{i4}x_{i4} + \gamma_{i5}x_{i5} + \gamma_{i6}x_{i6} + \gamma_{i0} \end{Bmatrix} \quad (6)$$

where y_j represents predictive tracking models of the PHIVP and PHIVN, and γ_{ij} is the premise parameter of the ANFIS model. The antecedent (Ψ_{ij}) fuzzy set ranges as linguistic terms {L: low value, ML: medium low, M: medium, MH: medium high, H: high, and VH: very high value}.

The ANFIS simulation was implemented using two data sets: training and testing. For training, the input data were used to create a matrix relationship and construct fuzzy MFs for each model. For each individual input data point, fuzzy sets (Ψ_{ij}) consisted of six values: L, ML, M, MH, H, and VH. Each fuzzy model has 6 MFs for each input data. The MFs and fuzzy sets corresponding to the fuzzy model of PHIVP and PHIVN were obtained from the training data, and are illustrated in **Figure 4(a)–(c).** The fuzzy sets and MFs were $\mu\Psi^{FR}{}_{ij}$, where $\mu_{SEXR}{}^{6}{}_{6i} = \{\mu_L, \mu_{ML}, \mu_M, \mu_{HM}, \mu_H, {}_{VH}\}$, $\mu_{FMA}{}^{6}{}_{6i} = \{\mu_L, \mu_{ML}, \mu_M, \mu_{HM}, \mu_H, {}_{VH}\}$, $\mu_{EDU}{}^{6}{}_{6i} = \{\mu_L, \mu_{ML}, \mu_M, \mu_{HM}, \mu_H, {}_{VH}\}$, $\mu_{INC}{}^{6}{}_{6i} = \{\mu_L, \mu_{ML}, \mu_M, \mu_{HM}, \mu_H, {}_{VH}\}$, $\mu_{ART}{}^{6}{}_{6i} = \{\mu_L, \mu_{ML}, \mu_M, \mu_{HM}, \mu_H, {}_{VH}\}$, $\mu_{\{HHIVP\ or\ HHIVN\}}{}^{6}{}_{6i} = \{\mu_L, \mu_{ML}, \mu_M, \mu_{HM}, \mu_H, {}_{VH}\}$ for the developed ANFIS models (PHIVP & PHIVN). The antecedents of the fuzzy rules are illustrated in **Tables A1, A2,** and the consequences of the models obtained from the ANFIS training procedure are represented in **Appendix III**. The fuzzy-rules explicit representation of PHIVP and PHIVN models based on Eq. (6), as shown in **Appendices IV** and **V**. Following 300 training data iterations (iter), the final developed models were optimized and validated by data testing. When the Root Mean Square Error (RMSE) reached the lowest value in both the training and testing data sets, which was found at 300 iterations, the models were determined to have met the requirements of well-based regression (R).

2.3. Simulation details of the FSC-APSO-ANFIS approach

2.3.1. Data loading and definition

146 districts and data from six datasets (SEXR, ART, FMA, EDU, INC, and HHIVP or HHIVN) were uploaded as described (x_i). About 85% of these datasets were utilized for training, and the remaining 15% were used for testing and data validation. The six fuzzy rules and their clusters are constructed using the parameters of k: number of clusters, C: number of cluster centers, α and β: acceleration parameters, N_p: number of particles (swarm), τ: number of iterations, and r_a: radius of clusters.

2.3.2. Parameters initialization for data clustering

Initialize the parameters r_a, α, β, and create a swarm N_p matrix for V, **Ω**, and gbest to partition the input datasets into six clusters. Identify the number of clusters, k', and set the density of data. Then, calculate the first cluster center 'C_1' to the last cluster center 'C_6'and update FR in accordance with the MFs. Each MF's cluster center is selected with the intention of dispersing the MFs over the entire range. Each 'C' value is selected at random within its corresponding range after the range is partitioned. Set the cluster radiuses, create an objective function-based RMSE, calculate the 'gbest', and update the particle's velocity 'V' and its position '**Ω**'. The 'V' values are generated at random for each iteration, and particle '**Ω**' is calculated for the optimal cluster centers using the cluster radius 'r_a'.

2.3.3. Predictive data modeling

The clustering data for 6 input data, each input has 146 datasets, was simulated into training and testing data. In this regard, 22 districts (15%) of the dataset were used to validate the prediction model (PHIVP and PHIVN, **Appendices IV and V**) that was created using the training data from 124 districts (85%). Based on two scenarios, the simulation's performance is assessed using the objective function, RMSE.

2.3.3.1. FSC-APSO Parameter setting

The FSC-APSO algorithm's settings are as follows: swarm = 50 particles, iterations = 300, cluster radius = 0.53; number of cluster centers = 6; and acceleration factors α and β were set at 0.48 and 0.65, respectively.

2.3.3.2. GNN Parameter setting

The GNN model's parameters include: number of layers = nodes = 3, number of input features = 18, number of total parameters = 14 representing (number of weights = 12 & number of biases = 2), random initialization values ranged between – 0.55 to + 0.55, number of iterations = 300.

2.3.3.3. ANFIS Parameter setting

The **ANFIS** model's parameters include neuron count = 93, fuzzy rule count = 6, linear parameters = 30, and nonlinear parameters = 42, number of iterations = 300. In all cases, these simulation parameters were applied.

3. Results

3.1. Predictive model performance

As a result, the tracking models of the all-data, training data, and testing data closely matched the actual data, as shown in **Figures 5(a)–(b)–(c)–(d)–(e)–(f).** Models' performance is assessed using an objective metric, such as the RMSE displayed in **Figures 5(g)–(h)**, where the lowest RMSE indicates the highest model regression and better accuracy. Thus, RMSE can be expressed in Eq. (7),

$$RMSE = \sqrt{\frac{(collected\ data - predictive\ data)^2}{N}} \tag{7}$$

where N =146 denotes the number of datasets, y_{act} = actual collected data and y_{pre} = predicted data. Additionally, the coefficient of determination (R^2) evaluates the overall model's goodness, which expresses expressed in Eq. (8), y_{mn} = data's mean.

$$R^2 = 1 - \sum_{i=1}^{N} \frac{(y_{act} - y_{pre})^2}{(y_{act} - y_{mn})^2} \tag{8}$$

Lower RMSE indicates higher predictive accuracy, while an R^2 evaluates good agreement between actual and predicted data. As seen in **Table 3**, Scenario I (FSC-APSO-ANFIS) performed better than comparable literature review studies when compared to the upgraded ANFIS (scenario II) and GNN (scenario III).

3.2. Comparative studies

We found that among people living with HIV aged 15 to 49 years across all 146 districts in Uganda, the estimated PHIVP individuals were 5.1%, and the PHIVN with estimated at 33,432 for the year 2023, based-actual data gathered from (https://www.mnypa.org/docs/1723095739.pdf) regarding HIV/AIDS facts. After accounting for influential variables among HIV-positive individuals, this

study tracked PHIVN with 33,296 out of a total of 33,432 instances and estimated that the median PHIVP among the same set of people was 4.92%. In this study, a total of 146 × 6 district datasets (6 input data, each with 124 datasets) were included. The validation-based testing data comprised **22** district datasets (**15%**), of which 124 datasets (**85%**) were used to formulate the predictive and tracking models for PHIVP and PHIVN, respectively. As seen in **Figures 5(a)–(b),** all datasets (100%) for PHIVP and PHIVN have been predicted in comparison with the scenarios (I) , (II), and obtained data, **Figures 5(c)–(d)** 85% training datasets for PHIVP and PHIVN have been predicted, tested in **Figures 5(e)–(f),** and evaluated with 300 iterations by RMSE in **Figures 5(g)–(h).** Following training datasets, the estimated and tracked models PHIVP and PHIVN exhibit *P*-values of <0.001 ($P < 0.001$) for scenario I, *P*-value = 0.001 ($P = 0.001$) for scenario II, and *P*-value = 0.001 ($P = 0.001$) for scenario III respectively. Using RMSE as an objective metric, the PHIVP–PHIVN were evaluated at 1.65–24.6, 1.76–32.4, and 1.77–34.6 by scenario I scenario II, and scenario III, respectively. With a validation of 85.1% (R = 0.851) and an error of RMSE (1.6553) in **Table 3,** the proposal (scenario I) demonstrated its capacity to predict PHIVP as shown in **Figures 54(a-c-e).** Simultaneously, the same method has tracked the PHIVN extremely well, as demonstrated in **Figures 5(b-d-f),** with a testing performance of 98.7% (R = 0.987) in **Table 3** and an error of RMSE in **Figure 5(h).** In the district-level-based scenario, I showed a lower RMSE for PHIVP and PHIVN. For every district in Uganda, scenario I showed better tracking of PHIVP and PHIVN than scenario II and scenario III. In particular, the regressions for all data of PHIVP and PHIVN in scenario I were higher (0.823 and 0.987, respectively) than in scenario II (0.820 and 0.979) and then in scenario III (0.771 and 0.941). A simulation's results demonstrated that the developed model-based scenario was a reliable and effective tool for predicting and tracking PHIVP and PHIVN. When comparing PHIVP and PHIVN with the obtained actual data, scenario I demonstrated its superiority over scenario II and scenario III.

3.3. Model analysis outcome

The optimal data for the PHIVP in this study are; the ART service was set to 83% (80–>86%), the SEXR was 0.24 (0.18–0.66), the average INC was \$242 (225–\$455), the EDU was 0.53 (0.5–0.67), the FMA was 0.47 (0.43–0.53), and the HHIVP or HHIVN were 4.8% (2.8–5.4%) or 656 (106–38,132), respectively. The PHIVP's median for each **group** was determined among 4 districts of Uganda (NR, ER, WR, & CR), see **Appendix I**. Three scenarios (I, II & III) in this observational study have been carried out and compared with the actual collected data, as illustrated in **Figures**

6(a)–(b)–(c)–(d). The prediction model shows that most PHIVP in the NR, WR, and CR in **Figures 6(a)–(c)–(d)** can estimate and track every district in those regions. Scenario I has consistently remained at or marginally above the actual PHIVP average in some groups (ER) shown in **Figure 6(b)**. However, when compared to the actual data collected, the results of scenarios I, II, and III in the ER regions were unable to track PHIVP in some districts. Scenario I's ability to follow PHIVNs in regions with high HIV incidence further highlights its advantages. Scenarios II and III monitored 33,948 and 34,561 new incidents; however, it showed 516 and 1129 outbreaks, respectively. The scenarios (II & III) in **Figure 6(e)** was unable to track certain districts, scenario I tracked the number of PHIVN with a reduction of 136 cases (0.36%) for all ages and 15–49 years.

4. Discussion

4.1. Key Findings

Using the Uganda AIDS Commission's 2023 datasets, three AI scenarios (models) were used in this work to forecast the spread of HIV (PHIVP) and new incidences (PHIVN) based on related parameters (eco, educational, awareness, therapy, sex, and historical HIV). The performance of the models in scenario III (GNN) [21] and scenario II (improved ANFIS) [43], which utilized the data clustering matrix with six input datasets, showed reasonable findings, while scenario I indicated that it was more effective in applications requiring adaptability for tracking PHIVP and PHIVN. Despite the low values in new incidences and HIV prevalence in the real-time environment, the results obtained by the scenarios (I, II, & III) showed a rise in new PHIVN and a high PHIVP% in the ER regions. In contrast to the other scenarios II and III (see Tables 2 & 3, Figure 6), the study model (scenario I) demonstrated improved tracking and higher accuracy in the NR, WR, and CR regions, despite an increase in PHIVP for some ER regions.

4.2. Key Findings Argument

We compare the statistical data gathered from (https://www.mnypa.org/docs/1723095739.pdf) with the findings of PHIVP and PHIVN training using three adopted scenarios (I, II, and III)-based AI techniques. As reported, the actual median annual **PHIVP** among individuals (15-49) years in the NR ranged from lower to higher rates in Nabilatuk–Culu City, at approximately 3.95% (0.5–10.5%), whereas the median PHIVP by scenarios I, scenario II, and scenario III was 3.85%, 4.05%, and 4.03%, respectively, as given in **Table 2.** While the median PHIVP under scenario I, scenario

II, and scenario III was 3.69%, 3.93%, and 3.94%, respectively, the actual median yearly of 2023 PHIVP in the ER in Pallisa–Soroti ranged from lower to higher, at approximately 3.14% (1.1–12.5%). In contrast, the districts of WR and CR had median PHIVP of 5.81% and 6.35% under scenarios I, 5.44% and 6.69% under scenarios II, and 5.46% and 6.58% under scenarios III respectively. Meanwhile, the actual median of PHIVP for people aging (15-49) in the districts of Bundibugyo–Fort Portal City and Buvuma–Kalangala in both regions varied from lower to higher, at approximately 5.89% (2.2–14.1%) and 7.28% (4.0–13.1%), respectively. In addition, scenarios II and III failed to track the decline in PHIVP in the ER, but they did lower the PHIVP in the other three regions. In general, the actual PHIVP is 5.1% (0.5–14.1%). Scenarios (I, II, and III) estimated the overall PHIVP in 146 districts to be reduced by (4.92%, 5.03, and 5.2%), respectively, and therefore, scenarios I and II reduced the PHIVP by 3.53% and 1.37%, respectively, and failed by III. **Figure 6** illustrates the tracking of PHIVN of all ages, and it is observed that the proposed model (scenario I in **Figure 6(e)–(f)–(g**) shows its ability to track every single district of each region and predict new incidence 'PHIVN'. While scenarios II and III were unable to track certain districts in WR and CR in **Figure 6(e)**. Scenario I reduced the number of PHIVNs by 136 cases (0.36%) for all ages and 15–49 years in **Figures 6(f)–(g)** after deducting 4700 of PHIVNs for those aged 0–14 years, while II and III were outreached beyond the actual data of new infections as shown in **Table 2**. The objective metric of RMSE was used to evaluate the overall performance index with 300 iterative processes for PHIVP and PHIVN models, as illustrated in **Figures 5(g) and 5(h),** respectively. The analysis using scenario I demonstrated a high tracking for new incidence, with 99.6% for all ages and those aged 15–49, see **Figure 6(h)**. With an outbreak of 516 and 1129 cases, scenario II and scenario III tracked the new incidence with 98.6% and 97.1%, respectively. For PHIVP, both scenarios (I & II) were performed well, estimating the PHIVP median, which matched the estimated statistical data by the UNAIDS Uganda HIV/AIDS Commission.

4.3. Study strengths

The study-based FSC-APSO-ANFIS (scenario I) represented a potent computational tool and exhibited great strength and accuracy, which makes it superior to the regression and statistical models with a high regression value. Furthermore, the study is deemed adaptable and appropriate for any health outbreaks/epidemics. With a score of (R = 85.3%) for overall PHIVP prediction and (R = 98.7%) for tracking 'PHIVN' across Uganda. To assess the robustness of our results,

sensitivity analyses were performed by varying the cluster radius and number of particles in the FSC-APSO optimization process, as well as by altering the training-to-testing data ratio from 85:15 to 80:20. These variations did not materially affect the model's predictive accuracy (changes in RMSE <5%), suggesting that the findings are stable across reasonable parameter modifications. The **study significantly** allows authorized individuals to take action based on the tracking model, particularly in districts with a high rate of PHIVN that contribute to elevated PHIVP.

4.4. Study limitations

For huge data, the proposed technique requires a long simulation time for training data, and when the number of iterations is small, the accuracy may be affected. While the sensitivity analyses were performed by altering the number of iterations from 300 to 200. The variations did materially affect the overall model performance (change in RMSE > 5, R = 0.812 for PHVIP and R = 0.954 for PHIVN); more iterations mean better accuracy. To improve accuracy and reduce performance time, strategies can be introduced. We recommend using a 5-layer ANFIS integrating with meta-heuristic algorithms.

4.5. Uganda's progress based on 95-95-95 UNAIDS targets

ART therapy is essential for reducing PHIVP and avoiding PHIVN. In Uganda, as of 2023, estimates 92% of all PLHIV (15-49) years were aware of their status, 83% were on ART, and 79% were virally suppressed https://www.mnypa.org/docs/1723095739.pdf. As of right now, about 80.1% of PLHIV know their status, 96% have received ART, and 92% are virally suppressed; these developments underscore the significance of looking beyond the HIV cascade. The UNAIDS established the 95-95-95 targets, which call for 95% of all PLHIV to be diagnosed, 95% of those diagnosed with ART to be treated, and 95% of those treated to have viral suppression by 2030 [51-53]. With this progress, Uganda is on track to meet its 95-95-95 targets by 2030. Ugandan officials continued their efforts to reduce HIV prevalence, meet UNAIDS targets, and end the HIV/AIDS epidemic by 2030 despite the difficulties caused by the current US administration's 24% USAID freeze (90 days) from SSA countries since January 2020, which has not yet resumed.

4.6. Generalizability

While this model was developed using Ugandan surveillance data, the methodological framework (FSC-APSO + ANFIS) is adaptable to other sub-Saharan African contexts or countries with similar

HIV epidemiological profiles. However, generalization should be approached cautiously, as socioeconomic and health system factors may differ substantially.

5. Conclusions

This study offers and provides a powerful predictive tool for projecting HIV prevalence and new incidences in adults 15+ years old by using treatment, socio-data, and historical HIV data collected from https://www.mnypa.org/docs/1723095739.pdf in 2023. The study developed a general computational model with adequate agreement that has a robust tracking system (PHVIP–PHIVN: training dataset 85% with R = 0.832–0.987; PHVIP–PHIVN: validation dataset 15% with R = 0.851–0.987). Calibrating the RMSE to 1.6553 and 24.629 for PHIVP and PHIVN, respectively, supports the precision of predictive modeling for changes with an error <5%. After data had been clustered, trained, and tested, the modeling study achieved a high accuracy with HIV (prevalence and new incidences) tracking of 99.6%. The modeling study reveals that it enhances data-driven decisions in addressing PLHIV in district regions where PHIVP and PHIVN are high. The findings of this study will be a baseline method for predicting the spread of HIV and tracking incidences in years to come. Also, it can help the authorities (lawmakers & health officials) in making decisions and policies in Uganda to meet UNAIDS targets and end the HIV/AIDS epidemic by 2030, such as implementing targeted prevention programs and increasing access to treatment for affected populations.

Acknowledgement

We are grateful to the Uganda AIDS Commission HIV for making PLHIV data publicly available on the website. Also, we are appreciative of everyone who provided survey data on individual income, awareness, and education to support our research. Our gratitude's to Princess Nourah bint Abdulrahman University Researchers (PNURSP2026R764), for their support.

Ethical statement

This study analyzed anonymized and publicly available HIV data; no personal identifiers were used, and ethical approval was therefore not required https://www.mnypa.org/docs/1723095739.pdf.

Declaration of competing interest

The authors declare no conflict of interest.

Funding

No funding was provided to the authors to support this study.

Data availability statement

The data collection https://www.mnypa.org/docs/1723095739.pdf, datasets, and data model generated from the collected data are publicly available by the corresponding author in the Appendix

Author contributions

Elnazeer Ali and Muna Elsadig: Conceptualization, Methodology, and Writing the Original Draft, Umi Omar, Val Hyginus, Salhi, and Yasreen Alkhalig: Data collection and analysis, and editing. Adel Mergani, Abuagla Dafalla, Sahla Ahmed, and Bienfait Vahwere: Writing review and data identification

References

[1] E. I. Obeagu and G. U. Obeagu, "Preventive measures against HIV among Uganda's youth: Strategies, implementation, and effectiveness," *Medicine,* vol. 103, p. e40317, 2024.

[2] S. Chidrawar, S. Sane, M. Mamulwar, S. Bembalkar, R. Thakur, T. Bhatnagar*, et al.*, "Effect of antiretroviral therapy on retention of people living with HIV in India (2012–2017): a retrospective, cohort study," *The Lancet Regional Health-Southeast Asia,* vol. 34, 2025.

[3] N. Hanum, M. Handayani, A. Padmasawitri, Z. Zazuli, K. Anggadiredja, M. N. Pohan*, et al.*, "HIV incidence and adherence after pre-exposure prophylaxis initiation in key populations in Indonesia: Findings from a real-world pilot program 2021-2023," *IJID regions,* vol. 14, p. 100573, 2025.

[4] C. Su, Y. Ma, H. Liang, A. Huang, W. Deng, J. Zhou*, et al.*, "ART and Serum albumin are influencing factors of the 5-year survival rate of people living with HIV undergoing maintenance hemodialysis caused by HIV: A cohort study," *Medicine,* vol. 102, p. e35494, 2023.

[5] I. Matovu and M. Musoke, "Associated Risk Factors of HIV Infection among Adolescent Girls and Young Women Seeking Services at a Tertiary Hospital in central Uganda," *Journal of Environmental Science and Public Health,* vol. 8, pp. 143-149, 2024.

[6] J. Ssemakula Moses, "Understanding Youth Vulnerability to HIV/AIDS in Uganda: Social, Economic, and Behavioural Dimensions," *MANAGEMENT (NIJCIAM),* vol. 4, 2024.

[7] I. Kasamba, J. Mugisha, A. Abaasa, R. Makanga, E. Ruzagira, P. Kaleebu*, et al.*, "Thirty years of change in HIV incidence among adults in the Kyamulibwa General Population Cohort in rural southwest Uganda, 1989-2021," *International journal of infectious diseases,* vol. 129, pp. 125-134, 2023.

[8] C. Kremer, A. Kamali, M. Kuteesa, J. Seeley, N. Hens, and R. N. Nsubuga, "Modelling the impact of combining HIV prevention interventions on HIV dynamics in fishing communities in Uganda," *BMC infectious diseases,* vol. 23, p. 173, 2023.

[9] R. H. Gray, F. Makumbi, D. Serwadda, T. Lutalo, F. Nalugoda, P. Opendi*, et al.*, "Limitations of rapid HIV-1 tests during screening for trials in Uganda: diagnostic test accuracy study," *Bmj,* vol. 335, p. 188, 2007.

[10] J. Ouma, C. Jeffery, J. J. Valadez, R. K. Wanyenze, J. Todd, and J. Levin, "Combining national survey with facility-based HIV testing data to obtain more accurate estimate of HIV prevalence in districts in Uganda," *BMC public health,* vol. 20, p. 379, 2020.

[11] M. Alehegn, "Application of machine learning and deep learning for the prediction of HIV/AIDS," *HIV & AIDS Review. International Journal of HIV-Related Problems,* vol. 21, pp. 17-23, 2022.

[12] Y. Xiang, J. Du, K. Fujimoto, F. Li, J. Schneider, and C. Tao, "Application of artificial intelligence and machine learning for HIV prevention interventions," *The Lancet HIV,* vol. 9, pp. e54-e62, 2022.

[13] R. Jin and L. Zhang, "AI applications in HIV research: advances and future directions," *Frontiers in Microbiology,* vol. 16, p. 1541942, 2025.

[14] M. Jaiteh, E. Phalane, Y. A. Shiferaw, H. Jallow, and R. N. Phaswana-Mafuya, "The application of machine learning algorithms to predict HIV testing using evidence from the 2002–2017 South African adult population-based surveys: an HIV testing predictive model," *Tropical Medicine and Infectious Disease,* vol. 10, p. 167, 2025.

[15] B. M. Rashed and N. Popescu, "medical image-based diagnosis using a hybrid adaptive neuro-fuzzy inferences system (ANFIS) optimized by GA with a deep network model for features extraction," *Mathematics,* vol. 12, p. 633, 2024.

[16] Y. Shen, T. Liu, J. Chen, X. Li, L. Liu, J. Shen*, et al.*, "Harnessing artificial intelligence to optimize long-term maintenance dosing for antiretroviral-naive adults with HIV-1 infection," *Advanced Therapeutics,* vol. 3, p. 1900114, 2020.

[17] A. Endawkie, B. A. Miheretu, A. Yalew, P. S. Nyasulu, G. Worku, A. Asaminew*, et al.*, "Machine learning approach and geospatial analysis to determine HIV infection, awareness status, and transmission knowledge among adults in Sub-Saharan Africa," *BMC Research Notes,* vol. 17, p. 379, 2024.

[18] A. M. Almarashi, "Statistical modelling and forecasting of HIV and anti-retroviral therapy cases by time-series and machine learning models," *Scientific Reports,* vol. 15, p. 27033, 2025.

[19] T. S. PLAN, "2023-2028."

[20] B. G. Williams, S. Gupta, M. Wollmers, and R. Granich, "Progress and prospects for the control of HIV and tuberculosis in South Africa: a dynamical modelling study," *The Lancet Public Health,* vol. 2, pp. e223-e230, 2017.

[21] E. Yu, J. Du, Y. Xiang, X. Hu, J. Feng, X. Luo*, et al.*, "Explainable artificial intelligence and domain adaptation for predicting HIV infection with graph neural networks," *Annals of Medicine,* vol. 56, p. 2407063, 2024.

[22] S. Nsanzimana, G. N. Rwibasira, S. S. Malamba, G. Musengimana, E. Kayirangwa, S. Jonnalagadda*, et al.*, "HIV incidence and prevalence among adults aged 15-64 years in Rwanda: Results from the Rwanda Population-based HIV Impact Assessment (RPHIA) and District-level Modeling, 2019," *International Journal of Infectious Diseases,* vol. 116, pp. 245-254, 2022.

[23] X. Tian, J. Chen, X. Wang, Y. Xie, X. Zhang, D. Han*, et al.*, "Global, regional, and national HIV/AIDS disease burden levels and trends in 1990–2019: a systematic analysis for the global burden of disease 2019 study," *Frontiers in Public Health,* vol. 11, p. 1068664, 2023.

[24] R. Banadakoppa Manjappa, P. Bhattacharjee, S. Y. N. Shaw, J. Gitonga, J. Kioko, F. Songok*, et al.*, "A sub-national HIV epidemic appraisal in Kenya: a new approach for identifying priority geographies, populations and programmes for optimizing coverage for HIV prevention," *Journal of the International AIDS Society,* vol. 27, p. e26245, 2024.

[25] E. L. Korenromp, K. Sabin, J. Stover, T. Brown, L. F. Johnson, R. Martin-Hughes*, et al.*, "New HIV infections among key populations and their partners in 2010 and 2022, by world region: a multisources estimation," *JAIDS Journal of Acquired Immune Deficiency Syndromes,* vol. 95, pp. e34-e45, 2024.

[26] T. Peng, X. Jin, C. Xiong, H. Juan, C. Juhai, W. Longhang*, et al.*, "Prevalence of sarcopenia and association with HIV infection in China elderly: An observational study," *Medicine,* vol. 103, p. e38532, 2024.

[27] N. A. Shabbir, S. B. Kant, K. Rashid, U. Hafeez, A. A. Akbar, S. W. Batool*, et al.*, "Prevalence of HIV/AIDS among pregnant women in North American region: A systematic review and meta-analysis," *Medicine,* vol. 103, p. e40339, 2024.

[28] E. I. Obeagu, I. Isiko, and G. U. Obeagu, "Immunological factors influencing HIV acquisition in infants: A narrative review," *Medicine,* vol. 104, p. e43039, 2025.

[29] C.-C. Lin, M.-C. Hsieh, H.-C. Hung, S.-M. Tsao, S.-C. Chen, H.-J. Yang*, et al.*, "Human papillomavirus prevalence and behavioral risk factors among HIV-infected and HIV-uninfected men who have sex with men in Taiwan," *Medicine,* vol. 97, p. e13201, 2018.

[30] S. Kizito, F. M. Ssewamala, T. B. Neilands, P. Nabunya, P. Namatovu, J. Nabayinda*, et al.*, "A prediction model for virologic failure in adolescents living with HIV in Uganda: Findings from the Suubi+ Adherence study," *Public Health,* vol. 244, p. 105753, 2025.

[31] H. Wang, T. M. Wolock, A. Carter, G. Nguyen, H. H. Kyu, E. Gakidou*, et al.*, "Estimates of global, regional, and national incidence, prevalence, and mortality of HIV, 1980–2015: the Global Burden of Disease Study 2015," *The lancet HIV,* vol. 3, pp. e361-e387, 2016.

[32] T. D. Frank, A. Carter, D. Jahagirdar, M. H. Biehl, D. Douwes-Schultz, S. L. Larson*, et al.*, "Global, regional, and national incidence, prevalence, and mortality of HIV, 1980–2017, and forecasts to 2030, for 195

countries and territories: a systematic analysis for the Global Burden of Diseases, Injuries, and Risk Factors Study 2017," *The lancet HIV,* vol. 6, pp. e831-e859, 2019.

[33] I. Taramusi, J. Stover, R. Glaubius, T. Apollo, G. Ncube, O. Mugurungi*, et al.*, "HIV incidence and prevalence projections for Zimbabwe: Findings from five mathematical models," *African Journal of AIDS Research,* vol. 24, pp. 44-52, 2025.

[34] E. Yaylali and Z. M. Erdogan, "Estimating HIV incidence in Türkiye: results from two mathematical models," *BMC Infectious Diseases,* vol. 25, p. 367, 2025.

[35] J. Ouma, C. Jeffery, C. A. Awor, A. Muruta, J. Musinguzi, R. K. Wanyenze*, et al.*, "Model-based small area estimation methods and precise district-level HIV prevalence estimates in Uganda," *PloS one,* vol. 16, p. e0253375, 2021.

[36] D. W. Currie, R. Apondi, C. A. West, S. Biraro, L. N. Wasula, P. Patel*, et al.*, "A comparison of two population-based household surveys in Uganda for assessment of violence against youth," *PLoS one,* vol. 16, p. e0260986, 2021.

[37] T. Hallett and G. Garnett, "Estimating incidence of HIV infection in Uganda," *Jama,* vol. 301, pp. 159-161, 2009.

[38] M. Farahani, W. L. Kirungi, S. M. Farley, V. Mugisha, D. Hoos, T. F. Smart*, et al.*, "Progress in the HIV response in Uganda: findings from two sequential population-based HIV impact assessment surveys, 2017–21," *The Lancet HIV,* vol. 12, pp. e568-e577, 2025.

[39] A. A. Kim, T. Hallett, J. Stover, E. Gouws, J. Musinguzi, P. K. Mureithi*, et al.*, "Estimating HIV incidence among adults in Kenya and Uganda: a systematic comparison of multiple methods," *PloS one,* vol. 6, p. e17535, 2011.

[40] J. Larmarange, "Uganda: Developing subnational estimates of HIV prevalence and the number of people living with HIV," Unaids, 2014.

[41] F.-s. Yuan, L. Liu, L.-h. Liu, Y.-l. Zeng, L.-l. Zhang, F. He*, et al.*, "Epidemiological and spatiotemporal analyses of HIV/AIDS prevalence among older adults in Sichuan, China between 2008 and 2019: a population-based study," *International Journal of Infectious Diseases,* vol. 105, pp. 769-775, 2021.

[42] N. Sandamini, N. Jayathilaka, L. Vincent, A. Ananthakumaran, S. Rasmy, and Y. D. Lee, "HIV Treatment and Cure: A Critical Review of Scientific Advances and Therapeutic," 2025.

[43] Purwanto, C. Eswaran, and R. Logeswaran, "Improved adaptive neuro-fuzzy inference system for HIV/AIDS time series prediction," in *International Conference on Informatics Engineering and Information Science*, 2011, pp. 1-13.

[44] E. A. H. ABDALLA, "Modeling and optimization of the industrial plant using artificial intelligent techniques," Universiti Teknologi PETRONAS, 2020.

[45] I. Valladolid-Acebes and A. W. Steven, "Prevalence of HIV and Antiretroviral Therapy (ART) Coverage in Uganda: A Comprehensive Study of 2022 Trends."

[46] D. Denardo, S. A. Mojola, E. Schatz, and F. X. Gómez-Olivé, "Antiretroviral therapy and aging as resources for managing and resisting HIV-related stigma in rural South Africa," *SSM-Qualitative Research in Health,* vol. 2, p. 100148, 2022.

[47] A. D. Haas, E. Radin, A. J. Hakim, A. Jahn, N. M. Philip, S. Jonnalagadda*, et al.*, "Prevalence of nonsuppressed viral load and associated factors among HIV-positive adults receiving antiretroviral therapy in Eswatini, Lesotho, Malawi, Zambia and Zimbabwe (2015 to 2017): results from population-based nationally representative surveys," *Journal of the International AIDS Society,* vol. 23, p. e25631, 2020.

[48] L. A. I. Olubayo, T. Mathema, C. Kabudula, L. K. Micklesfield, S. F. Mohamed, I. Kisiangani*, et al.*, "The prevalence, incidence, and sociodemographic risk factors of HIV among older adults in sub-Saharan Africa (AWI-Gen): a multicentre, longitudinal cohort study," *The Lancet Healthy Longevity,* vol. 6, 2025.

[49] F. E. A. James P.M. Ntozi, "Prospects for Fertility Decline in the Face of HIV/AIDS in Uganda," *https://www.un.org/development/desa/pd/sites/www.un.org.development.desa.pd/files/undp_egm_200107_paper_prospects_for_fertility_decline_in_the_face_of_hiv_aids_in_uganda_ntozi_ahimbisibwe.pdf,* 2001.

[50] E. A. H. Abdalla, M. Kumar, I. I. Abdalla, S. E. G. Mohamed, A. M. Soomro, M. Irfan*, et al.*, "Modeling and optimization of isolated combined heat and power microgrid for managing Universiti Teknologi PETRONAS energy," *IEEE Access,* vol. 11, pp. 74388-74409, 2023.

[51] K. Heath, J. Levi, and A. Hill, "The Joint United Nations Programme on HIV/AIDS 95–95–95 targets: worldwide clinical and cost benefits of generic manufacture," *Aids,* vol. 35, pp. S197-S203, 2021.

[52] R. E. Magobo, M. Mabaso, S. Jooste, L. Molopa, I. Naidoo, L. Simbayi*, et al.*, "95-95-95 HIV indicators among children younger than 15 years in South Africa: results from the 2017 national HIV prevalence, incidence, behaviour, and communication survey," *AIDS Research and Therapy,* vol. 22, p. 6, 2025.

[53] L. Frescura, P. Godfrey-Faussett, A. Feizzadeh, W. El-Sadr, O. Syarif, P. D. Ghys, *et al.*, "Achieving the 95 95 95 targets for all: A pathway to ending AIDS," *PloS one,* vol. 17, p. e0272405, 2022.

Figure Captions

Figure 1 shows the flowchart of district-level 2023 HIV data selection and analysis by using the FSC-APSO-ANFIS (denoted as scenario I) technique. Also, the flowchart includes the improved ANFIS (scenario II) and GNN (scenario III) approaches. This flowchart describes how the simulation implemented datasets for the predictive tracking PHIVP and PHIVN system for all 146 Ugandan districts, Facts on HIV/AIDS in Uganda 2023. Each model (PHIVP / PHIVN) has 6 variable inputs with 6 clusters (C_i)

Figure 2 illustrates 6 inputs–outputs diagram of the optimized four-layer **ANFIS** structure with two-intelligent levels. Level I employed the FSC-APSO algorithm to divide and group datasets into clusters (Ψ_{ij}), while level II used ANFIS for building a predictive–tracking model (y_i). **Layer 1** is a linguistic input (x_i), **layer 2** is a linear polynomial of input data with weights (w_i), **layer 3** is used to create fuzzy rules, and **layer 4** is the final output for the predictive PHIVP and PHIVN models. **FSC** is fuzzy subtractive clustering, **APSO** is accelerated particle swarm optimization, **ANFIS** is an adaptive neuro-fuzzy inference system.

Figure 3 illustrates data modeling: Figures (a)–(b) six fuzzy sets and memberships–six optimal clustering centers of HIV prevalence "PHIVP", Figures (c)–(d) six fuzzy sets and memberships–six optimal cluster centers of new incidence "PHIVN". Fuzzy sets in Figures (a)–(c) have different values ranging from low (L), medium low (ML), medium (M), medium high (MH), high (H), and very high (VH) with a degree of membership (MEM). The FSC-APSO algorithm adjusted the fuzzy sets into 6 cluster centers in Figures (b)–(d) to evaluate the PHIVP and PHIV.

Figure 4 shows data modeling: Figures (a)–(b): all districts predictive-tracking evaluated by the regression (R), Figures (c)–(d): 85% training predictive-tracking, Figures (e)–(f): 15% testing predictive-tracking, and Figures (g)–(h): objective metric as a RMSE for predictive-tracking model of PHIVP and PHIVN, respectively. It compares the all data to 3 scenarios of the PHIVP & PHIVN model using objective performance-RMSE. The model trained and tested with the datasets with 300 iterations, where the models showed low errors. Scenario I gave lower RMSE values (RMSE=1.657, 24.629) with $p < 0.001$ than Scenario II (1.761, 32.457) and scenario III (1.776, 34.629) with $p = 0.001$ for HIV prevalence and new infections, respectively.
RMSE: root mean square error, p is the probability of observing a test statistic

Scn I (scenario): Scn II (scenario II), Scn III (scenario III)

Scenario I (FSC-APSO-ANFIS), Scenario II (Improved ANFIS), and Scenario III (GNN)

Figure 5 illustrates the prediction's median: Figures (a)–(b)–(c)–(d): HIV prevalence (PHIVP) among people aged 15 – 49 years in NR, ER, WR, and CR, respectively; and Figures (e)–(f)–(g)–(h): median new HIV incidence by regional groups, all ages, 15–49 years, and tracking percentage, respectively.

(a) PHIVP median for the NR of 41 districts that represents 6 groups
(b) PHIVP median for the ER of 40 districts that represents 6 groups
(c) PHIVP median for the WR of 38 districts that represents 6 groups
(d) PHIVP median for the CR of 27 districts that represents 6 groups
(e) Tracking PHIVN for each region (NR, ER, WR, CR) by data collection vs. scenario I, scenario II, and scenario III
(f) Tracking PHIVN for all regions by data collection vs. scenario I vs. scenario II vs. scenario III
(g) Tracking PHIVN throughout Uganda (all four regions) among 15-49 years by data collection vs. scenario I vs. scenario II vs. scenario III
(h) Percentage tracking PHIVN throughout Uganda (all four regions) for 15-49 years and all ages by data collection vs. scenario I vs. scenario II vs. scenario III

Figure Legends

Figure 1. The proposed study's framework for predicting and tracking and PHIVP and PHIVN.

Figure 2. Inputs–outputs: HIV prevalence and new infections using improved ANFIS.

Figure 3. Data modeling: (a)–(b) fuzzy sets & memberships–optimal clustering centers of PHIVP, (c)–(d) fuzzy sets & memberships–optimal clustering centers of new PHIVN.

Figure 4. Prediction of PHIVP and tracking PHIVN: all data, testing data, and RMSE

Figure 5. The median prediction: PHIVP and all PHIVN in 146 districts for individuals aged 15 to 49.

Table Legends

Table 1: an overview of HIV incidence and prevalence using various approaches.

Table 2: PHIVP and PHIVN in Uganda's four administrative regions (146 districts) for 2023.

Table 3: Performance metric for PHIVP and PHIVN in Uganda's four administrative regions (146 districts) for 2023.

Table A1: The antecedent of six fuzzy rules for the PHIVP model

Table A2: The antecedent of six fuzzy rules for the PHIVN Model.

Appendices

turnitin Page 26 of 26 - AI Writing Submission Submission ID trn:oid:::1:3612769924
