# Supplementary material for: Artificial neuro-fuzzy intelligence for dynamic tracking and prediction of HIV prevalence and new infections among individuals aged 15 to 49 years in Uganda: Computational national-level models
Source: Medicine (Baltimore). 2026 Sep 18;105(38):e50760. doi: 10.1097/MD.0000000000050760 (PMC13593017; doi:10.1097/MD.0000000000050760)
Supplement: Supplementary file 2 [file medi-105-e50760-s001.docx]

**TS 1:** The antecedents of six fuzzy rules for the PHIVP model

| **SEXR*_i_*** | **ART*_i_*** | **FMA*_i_*** | **INC*_i_*** | **EDU*_i_*** | **HHIVP*_i_*** | **PHIVP** |
| --- | --- | --- | --- | --- | --- | --- |
| L  L  L  L  ML  L | MH  H  MH  MH  H  H | M  M  MH  MH  M  M | MH  VH  M  H  MH  M | MH  MH  M  M  M  M | ML  ML  ML  L  L  L | PHIVP1  PHIVP2  PHIVP3  PHIVP4  PHIVP5  PHIVP6 |

Table A1 gives the antecedent of six (6) fuzzy rules for the HIV prevalence (PHIVP) model.

**The abbreviations denote the following**:

L = low value

ML = medium low value

M = medium value

MH = medium high value

H = high value

**Inputs**

SEXR = sex rate

ART = Antiretroviral Therapy

FMA = family member awareness

INC = individual income

EDU = educational level

HHIVP = historical HIV prevalence
