# Supplementary material for: Artificial neuro-fuzzy intelligence for dynamic tracking and prediction of HIV prevalence and new infections among individuals aged 15 to 49 years in Uganda: Computational national-level models
Source: Medicine (Baltimore). 2026 Sep 18;105(38):e50760. doi: 10.1097/MD.0000000000050760 (PMC13593017; doi:10.1097/MD.0000000000050760)
Supplement: Supplementary file 4 [file medi-105-e50760-s003.docx]

*Appendix I: Administrative regions of Uganda*

There are four administrative regions: the northern region (NR), eastern region (ER), western region (WR), and central region (CR), ^[45].^ The following districts, NR represents 41 districts: (Abim, Adjumani, Agago, Alebtong, Amolatar, Amudat, Amuru) as group1, (Apac, Arua, Arua City, Dokolo, Gulu, Gulu City, Kaabong) as group2, (Karenga, Kitgum, Koboko, Kole, Kotido, Kwania, Lamwo) as group3, (Lira, Lira City, Madi Akollo, Maracha, Moroto, Moyo, Nabilatuk) as group4, (Nakapiripirit, Napak, Nebbi, Nwoya, Obongi, Omoro, Otuke) as group5, and (Oyam, Pader, Pakwach, Terego, Yumbe, Zombo) as group6.

The districts that represent the **ER** are: (Amuria, Budaka, Bududa, Bugiri, Bugweri, Bukedea, Bukwo) as group1, (Bulambuli, Busia, Butaleja, Butebo, Buyende, Iganga, Jinja) as group2, (Jinja City, Kaberamaido, Kalaki, Kaliro, Kamuli, Kapchorwa, Kapelebyong) as group3, (Katakwi, Kibuku, Kumi, Kween, Luuka, Manafwa, Mayuge) as group4, (Mbale, Mbale City, Namayingo, Namisindwa, Namutumba, Ngora) as group5, and (Pallisa, Serere, Sironko, Soroti, Soroti City, Tororo) as group6.

However, the **WR** is represented by the following: (Buhweju, Buliisa, Bundibugyo, Bunyangbu, Bushenyi, Fort Portal City, Hoima) as group1, (Hoima City, Ibanda, Isingiro, Kabale, Kabarole, Kagadi, Kakumiro) as group2, (Kamwenge, Kanungu, Kasese, Kazo, Kibaale, Ikuube) as group3, (Kiruhura, Kiryandongo, Kisoro, Kitagwenda, Kyegewa, Kyenjojo) as group4, (Masindi, Mbarara, Mbarara City, Mitooma, Ntoroko, Ntungamo) as group5, and (Rubanda, Rubirizi, Rukiga, Rukungiri, Rwampara, Sheema) as group6.

**CR** represents (Buikwe, Bukomansimbi, Butambala, Buvuma, Gomba) as group1, (Kalangala, Kalungu, Kampala, Kassanda, Kayunga) as group2, (Kiboga, Kyankwanzi, Kyotera, Luwero, Lwengo) as group3, (Lyantonde, Masaka, Masaka City, Mityana) as group4, (Mpigi, Mubende, Mukono, Nakaseke) as group5, and (Nakasongola, Rakai, Ssembabule, Wakiso) as group6. There are six groups in each administrative region, with four to seven districts representing each group. All 146 districts of Uganda are available on: <https://www.mnypa.org/docs/1723095739.pdf>.

[45]	I. Valladolid-Acebes and A. W. Steven, "Prevalence of HIV and Antiretroviral Therapy (ART) Coverage in Uganda: A Comprehensive Study of 2022 Trends."
