# Supplementary material for: Artificial neuro-fuzzy intelligence for dynamic tracking and prediction of HIV prevalence and new infections among individuals aged 15 to 49 years in Uganda: Computational national-level models
Source: Medicine (Baltimore). 2026 Sep 18;105(38):e50760. doi: 10.1097/MD.0000000000050760 (PMC13593017; doi:10.1097/MD.0000000000050760)
Supplement: Supplementary file 5 [file medi-105-e50760-s004.docx]

*Appendix II: ANFIS layers mathematical models*

Layer1: in this layer, each node represents a linguistic input using a Gaussian MEM, the output of this node represents a FR antecedent. The clusters and modules{[Q_11_(x_1_), …, Q_16_(x_1_)], [Q_21_(x_2_), …, Q_26_(x_2_)], [Q_31_(x_3_), …, Ψ_36_(x_3_)], [Ψ_41_(x_3_), …, Ψ_46_(x_4_)], [Ψ_51_(x_5_), …, Ψ_56_(x_5_)], [Ψ_61_(x_6_), …, Ψ_66_(x_6_)]} represent linguistic terms (L, ML, M, MH, H, VH) of the data x*_i_* = x*_1_*, x*_2_*, …., x*_n_*. The layer 1 output of the node *i* of each input can be expressed by,

${\mu\Psi}_{ij}(x_{i})=exp\left\{ \frac{{-(q_{l}-c_{i})}^{2}}{2\sigma_{i}^{2}} \right\}$	(A1)

where μ is the MF of each FR, q_l_ is the input data to the node at layer 1, c_i_ and 𝜎_i_ are MFs premise parameters of mean (cluster-center) and standard deviation, respectively. The premise parameters can be calculated based on the input data using FSC technique.

Layer 2: this layer represents a linear polynomial of the input data with the weights (w) of its terms which is denoted as coefficients. The output node (Π) of this layer is the product of MF computed as in Eq. (A2),

Layer 3: in this layer, the rule *i* is normalized by Eq. (A3)

$w_{i}=\prod_{i=1}^{i=6} {\mu\Psi}_{i,j}^{FR}\left( x_{i} \right), i=1, 2,\ldots.,6$	(A2)

$ѿ_{i}= w_{i}/\sum_{i=1}^{n} w_{l}$	(A3)

where ѿ is the weight of consequent of fuzzy rules w*_l_* = w_1_ + w_2_ + w_3_ + w_4_ + w_5_ + w_6_ = 1. After the calculation of the consequent weights of each FR. The output of the layer node (N) can be written in Eq. (A4), where γ_ij_ are the fuzzy consequent parameters

$ѿ_{i}y_{i}= ѿ_{i}\left\{ \begin{aligned} \gamma_{i1}x_{i1} +\gamma_{i2}x_{i2}+ \gamma_{i3}x_{i3}+ \gamma_{i4}x_{i4}+ \gamma_{i5}x_{i5}+ \gamma_{i6}x_{i6}+ \gamma_{i0}, \\ \gamma_{i1}x_{i1} +\gamma_{i2}x_{i2}+ \gamma_{i3}x_{i3}+ \gamma_{i4}x_{i4}+ \gamma_{i5}x_{i5}+ \gamma_{i6}x_{i6}+ \gamma_{i0} \\ \gamma_{i1}x_{i1} +\gamma_{i2}x_{i2}+ \gamma_{i3}x_{i3}+ \gamma_{i4}x_{i4}+ \gamma_{i5}x_{i5}+ \gamma_{i6}x_{i6}+ \gamma_{i0} \\ \gamma_{i1}x_{i1} +\gamma_{i2}x_{i2}+ \gamma_{i3}x_{i3}+ \gamma_{i4}x_{i4}+ \gamma_{i5}x_{i5}+ \gamma_{i6}x_{i6}+ \gamma_{i0} \\ \gamma_{i1}x_{i1} +\gamma_{i2}x_{i2}+ \gamma_{i3}x_{i3}+ \gamma_{i4}x_{i4}+ \gamma_{i5}x_{i5}+ \gamma_{i6}x_{i6}+ \gamma_{i0} \\ \gamma_{i1}x_{i1} +\gamma_{i2}x_{i2}+ \gamma_{i3}x_{i3}+ \gamma_{i4}x_{i4}+ \gamma_{i5}x_{i5}+ \gamma_{i6}x_{i6}+ \gamma_{i0} \end{aligned} \right\}$	(A4)

Layer 4: Equation (A4) can be represented as the final output for the predictive model is used to build FR as a first-order linear polynomial, which can be given in Appendix III and IV.
