# Supplementary material for: Artificial neuro-fuzzy intelligence for dynamic tracking and prediction of HIV prevalence and new infections among individuals aged 15 to 49 years in Uganda: Computational national-level models
Source: Medicine (Baltimore). 2026 Sep 18;105(38):e50760. doi: 10.1097/MD.0000000000050760 (PMC13593017; doi:10.1097/MD.0000000000050760)
Supplement: Supplementary file 6 [file medi-105-e50760-s005.docx]

*Appendix III: Formulation of optimal ANFIS for PHIVP computational models*

The explicit representation of fuzzy rules derived from ANFIS models for the HIV prevalence (PHIVP) that were clustered and optimized by the FSC-APSO algorithm*:*

$$FR1:iF \left\{ \begin{aligned} \left( SEXR is L \right)\mathrm{and}\left( ART is MH \right)\mathrm{and}\left( FMA is M \right)\mathrm{and}\left( INC is MH \right)\mathrm{and}\left( EDU is MH \right)\mathrm{and} \\ (HHIVP is ML) \end{aligned} \right\}\mathrm{Then}$$

$$PHIVP1 (y_{1})= \left\{ \begin{aligned} 0.1471\times SEXR - 1.3296\times ART+ 6.0783\times FMA+ 0.2727\times INC + \\ 2.8581\times EDU + 0.4258\times HHIVP + 20.0878 \end{aligned} \right\}$$

$$FR2:iF \left\{ \begin{aligned} \left( SEXR is L \right)\mathrm{and}\left( ART is H \right)\mathrm{and}\left( FMA is M \right)\mathrm{and}\left( INC is VH \right)\mathrm{and}\left( EDU is MH \right) \\ and (HHIVP_{(k-1)} is ML) \end{aligned} \right\}\mathrm{Then}$$

$$PHIVP2 (y_{2})= \left\{ \begin{aligned} 0.0124\times SEXR + 16.0276\times ART - 6.8246\times FMA- 0.1625\times INC + \\ 256.98\times EDU+ 1.8401\times HHIVP + 222.701 \end{aligned} \right\}$$

$$FR3:iF \left\{ \begin{aligned} \left( SEXR is L \right)\mathrm{and}\left( ART is MH \right)\mathrm{and}\left( FMA is MH \right)\mathrm{and}\left( INC is M \right)\mathrm{and}\left( EDU is H \right) \\ and (HHIVP is ML) \end{aligned} \right\}\mathrm{Then}$$

$$PHIVP3 (y_{3})= \left\{ \begin{aligned} -0.0298\times SEXR + 3.6042\times ART - 7.1169\times FMA-0.0469\times INC- \\ 15.18\times EDU - 0.0644\times HHIVP+ 70.8698 \end{aligned} \right\}$$

$$FR4:iF \left\{ \begin{aligned} \left( SEXR is L \right)\mathrm{and}\left( ART is MH \right)\mathrm{and}\left( FMA is MH \right)\mathrm{and}\left( INC is H \right)\mathrm{and}\left( EDU is M \right) \\ and (HHIVP is L) \end{aligned} \right\}\mathrm{Then}$$

$$PHIVP4 (y_{4})= \left\{ \begin{aligned} -2.8611\times SEXR + 21.9864\times ART + 1.5969\times FMA + 0.0195\times INC + \\ 0.1361\times EDU + 0.0823\times HHIVP + 0.643 \end{aligned} \right\}$$

$$FR5:iF \left\{ \begin{aligned} \left( SEXR is ML \right)\mathrm{and}\left( ART is H \right)\mathrm{and}\left( FMA is M \right)\mathrm{and}\left( INC is MH \right)\mathrm{and}\left( EDU is M \right) \\ and (HHIVP is L) \end{aligned} \right\}\mathrm{Then}$$

$$PHIVP5 (y_{5})= \left\{ \begin{aligned} 0.0288\times SEXR - 0.6583\times ART + 7.0525\times FMA + 0.6407\times INC \\ - 2.8839\times EDU + 1.8803\times HHIVP + 55.7075 \end{aligned} \right\}$$

$$FR6:iF \left\{ \begin{aligned} \left( SEXR is L \right)\mathrm{and}\left( ART is H \right)\mathrm{and}\left( FMA is M \right)\mathrm{and}\left( INC is M \right)\mathrm{and}\left( EDU is M \right) \\ and (HHIVP is L) \end{aligned} \right\}\mathrm{Then}$$

$PHIVP6 (y_{6})= \left\{ \begin{aligned} 0.0317\times SEXR + 5.8852\times ART- 10.132\times FMA + 12.8711\times INC - \\ 5.4401\times EDU + 12.177\times HHIVP+ 91.8075 \end{aligned} \right\}$
