# Supplementary material for: Artificial neuro-fuzzy intelligence for dynamic tracking and prediction of HIV prevalence and new infections among individuals aged 15 to 49 years in Uganda: Computational national-level models
Source: Medicine (Baltimore). 2026 Sep 18;105(38):e50760. doi: 10.1097/MD.0000000000050760 (PMC13593017; doi:10.1097/MD.0000000000050760)
Supplement: Supplementary file 7 [file medi-105-e50760-s006.docx]

*Appendix IV: Formulation of optimal ANFIS for PHIVN computational models*

The explicit representation of fuzzy rules derived from ANFIS models for the new HIV infection (PHIVN) that clustered-optimized by FSC-APSO algorithm:

$$FR1:iF \left\{ \begin{aligned} \left( SEXR is L \right)\mathrm{and}\left( ART is MH \right)\mathrm{and}\left( FMA is MH \right)\mathrm{and}\left( INC is MH \right)\mathrm{and}\left( EDU is MH \right)\mathrm{and} \\ (HHIVN is L) \end{aligned} \right\}\mathrm{Then}$$

$$PHIVN1 (y_{1})= \left\{ \begin{aligned} 0.0363\times SEXR- 0.0165\times ART + 9.7731\times FMA- 0.0219\times INC + \\ 1.7341\times EDU + 2.3569\times HHIVN + 3.0295 \end{aligned} \right\}$$

$$FR2:iF \left\{ \begin{aligned} \left( SEXR is L \right)\mathrm{and}\left( ART is H \right)\mathrm{and}\left( FMA is M \right)\mathrm{and}\left( INC is M \right)\mathrm{and}\left( EDU is M \right) \\ and (HHIVN is ML) \end{aligned} \right\}\mathrm{Then}$$

$$PHIVN2 (y_{2})= \left\{ \begin{aligned} -0.0017\times SEXR + 0.7718\times ART +3.6778\times FMA- 0.0126\times INC \\ - 0.5443\times EDU- 0.5268\times HHIVN- 47.984 \end{aligned} \right\}$$

$$FR3:iF \left\{ \begin{aligned} \left( SEXR is L \right)\mathrm{and}\left( ART is MH \right)\mathrm{and}\left( FMA is M \right)\mathrm{and}\left( INC is M \right)\mathrm{and}\left( EDU is M \right) \\ and (HHIVN is L) \end{aligned} \right\}\mathrm{Then}$$

$$PHIVN3 (y_{3})= \left\{ \begin{aligned} -0.8462\times SEXR+ 0.4433\times ART- 1.1362\times FMA + 0.0032\times INC \\ - 2.5795\times EDU + 0.4538\times HHIVN - 31.719 \end{aligned} \right\}$$

$$FR4:iF \left\{ \begin{aligned} \left( SEXR is ML \right)\mathrm{and}\left( ART is H \right)\mathrm{and}\left( FMA is M \right)\mathrm{and}\left( INC is MH \right)\mathrm{and}\left( EDU is M \right) \\ and (HHIVN is L) \end{aligned} \right\}\mathrm{Then}$$

$$PHIVN4 (y_{4})= \left\{ \begin{aligned} 0.2128\times SEXR- 0.1271\times ART + 0.3091\times FMA + 0.0286\times INC \\ + 0.2334\times EDU + 0.8188\times HHIVN + 0.0975 \end{aligned} \right\}$$

$$FR5:iF \left\{ \begin{aligned} \left( SEXR is L \right)\mathrm{and}\left( ART is MH \right)\mathrm{and}\left( FMA is MH \right)\mathrm{and}\left( INC is H \right)\mathrm{and}\left( EDU is M \right) \\ and (HHIVN is ML) \end{aligned} \right\}\mathrm{Then}$$

$$PHIVN5 (y_{5})= \left\{ \begin{aligned} 0.6651\times SEXR +0.0335\times ART + 0.0168\times FMA + 0.0187\times INC \\ + 0.0038\times EDU+ 0.0313\times HHIVN+ 2.0762 \end{aligned} \right\}$$

$$FR6:iF \left\{ \begin{aligned} \left( SEXR is L \right)\mathrm{and}\left( ART is H \right)\mathrm{and}\left( FMA is M \right)\mathrm{and}\left( INC is VH \right)\mathrm{and}\left( EDU is MH \right) \\ and (HHIVN is ML) \end{aligned} \right\}\mathrm{Then}$$

$$PHIVN6 (y_{6})= \left\{ \begin{aligned} -0.0564\times SEXR-0.0066\times ART + 0.1004\times FMA + 0.0059\times INC \\ + 1.5212\times EDU + 0.1261\times HHIVN + 6.8557 \end{aligned} \right\}$$
