# Supplementary material for: Artificial neuro-fuzzy intelligence for dynamic tracking and prediction of HIV prevalence and new infections among individuals aged 15 to 49 years in Uganda: Computational national-level models
Source: Medicine (Baltimore). 2026 Sep 18;105(38):e50760. doi: 10.1097/MD.0000000000050760 (PMC13593017; doi:10.1097/MD.0000000000050760)
Supplement: Supplementary file 8 [file medi-105-e50760-s008.docx]

| **Section/Topic** | **Item** | **Checklist Item** | **Page** |
| --- | --- | --- | --- |
| **Title and abstract** | | | |
| Title | 1 | Identify the study as developing and/or validating a multivariable prediction model, the target population, and the outcome to be predicted.   - Title: Artificial neuro-fuzzy intelligence for dynamic tracking and prediction of HIV prevalence and new infections among individuals aged 15 – 49 years in Uganda: Computational national-level models. - Identify the study as developing and validating a prediction model of HIV prevalence and incidences, the target people living with HIV, and the outcome to be tracked and projected. | 1 |
| Abstract | 2 | Provide a summary of objectives, study design, setting, participants, sample size, predictors, outcome, statistical analysis, results, and conclusions.   - The observational study of a predictive model was done by providing a study background objectives, study design, targeted people aged 15-49 years, districts data size, neuro-fuzzy predictor, study outcome, statistical analysis, findings (results), and conclusions. | 2 |
| **Introduction** | | | |
| Background and objectives | 3a | Explain the medical context (including whether diagnostic or prognostic) and rationale for developing or validating the multivariable prediction model, including references to existing models.   - Overviewing the studying and reviewing the previous literature review related to HIV prevalence and new HIV infections, - Identifying the research gap and the study’s main goal (solutions) by highlighting the potential approach for developing artificial neuro-fuzzy intelligence for predicting model, including references to existing models. | 3-10 |
|  | 3b | Specify the objectives, including whether the study describes the development or validation of the model or both.   - The main objective was to develop a computational model for predicting and tracking HIV prevalence and new infections for adults aged 15-49 years with a validation using existing actual data with generalizability. | 2 |
| **Methods** | | | |
| Source of data | 4a | Describe the study design or source of data (e.g., randomized trial, cohort, or registry data), separately for the development and validation data sets, if applicable.   - Quantify the source of data only (e.g., randomized data, survey, and collected data), separately for the development and validation data sets, if applicable. Quantify the data by recording of:  1. Records number = 146 SEXR ratio datasets (randomized data) 2. Records number = 146 ART datasets (available at the link (page 7) 3. Records number = 146 INC average datasets (survey then randomized) 4. Records number = 146 EDU ratio datasets (survey then randomized) 5. Records number = 146 FMA ratio datasets (survey then randomized) 6. Records number = 146 HHIVP datasets (available at the link, page 7) 7. Records number = 146 HHIVN datasets (available at the link (page 7)   The datasets were used 85% for training the predictive model, while the remaining 15% was for testing and validating the model. | 7 |
|  | 4b | Specify the key study dates, including start of accrual; end of accrual; and, if applicable, end of follow-up.   - The study is a predictive model by using 2023 data from Uganda Commissions AIDS available at the link in the manuscript page (7) | 7 |
| Participants | 5a | Specify key elements of the study setting (e.g., primary care, secondary care, general population) including number and location of centres.   - The study was carried out using data sources in section appendices. The outcome of the observation study has key elements such as datasets across each district, data representation, and number of datasets as summarized in the Figure 4 file. | 7 |
|  | 5b | Describe eligibility criteria for participants.   - irrelevant | - |
|  | 5c | Give details of treatments received, if relevant.   - irrelevant | - |
| Outcome | 6a | Clearly define the outcome that is predicted by the prediction model, including how and when assessed. (How & When)   - Define the predictive models for PHIVP & PHIVN - Method: Artificial intelligence-based improved ANFIS was used (collected data, surveys, and random data) to determine the outcome of the prediction. - The recorded data include 6x146 datasets for PHIVP and 6x146 datasets for PHIVN for 2023 for 146 districts. - Outcome: The collected data of HIV were determined and evaluated for each district's HIV prevalence and new HIV infections. | 8-13 |
|  | 6b | Report any actions to blind assessment of the outcome to be predicted.   - Irrelevant | - |
| Predictors | 7a | Clearly define all predictors used in developing or validating the multivariable prediction model, including how and when they were measured.   - The predictive models included sex rate ratio, educational level, individual income, awareness, ART therapy, and the historical HIV data of prevalence and incidences, found in Figure 2 file. **How:** Data collected via survey, randomly, and collected from Uganda AIDS commissions available on their website. Each predictor is detailed with 6 input data and measured **when** each district's data is entered to predict its HIV prevalence and incidences for 2023. | 7 |
|  | 7b | Report any actions to blind assessment of predictors for the outcome and other predictors.   - Irrelevant | - |
| Sample size | 8 | Explain how the study size was arrived at.   - Each model employed the predictive study's data size (6x146) to predict and project a proportion with performance-based regression (R) of 0.987 and 0.851, respectively, with errors of 0.013 and 0.149 for PHIVP and PHIVN as depicted in Table 3 file | 1 |
| Missing data | 9 | Describe how missing data were handled (e.g., complete-case analysis, single imputation, multiple imputation) with details of any imputation method.   - The predictive models for HIV prevalence and incidences incorporated all datasets or data sizes, with no missing or excluded data. | - |
| Statistical analysis methods | 10a | Describe how predictors were handled in the analyses.   - The data has been grouped, simulated, and trained for model prediction supported with statistics-based RMSE and regression. Table 3, Appendix III, IV, | 12-14 |
|  | 10b | Specify type of model, all model-building procedures (including any predictor selection), and method for internal validation.   - A hybrid of dynamic and linear models based on the linear and non-linear parameters of the prediction model. The final outcomes of the predictive models were validated and verified using the ANFIS technique. Refer to Appendices III and IV. | -- |
|  | 10d | Specify all measures used to assess model performance and, if relevant, to compare multiple models.   - Coefficient of determination was used to evaluate the model's performance and determine the outcome's variation proportion. Regression, RMSE, and p-value evaluated the models; the outcome was compared with previous similar studies and validated using actual data. See Table 3 | - |
| Risk groups | 11 | Provide details on how risk groups were created, if done**.**   - Not done in this study | - |
| **Results** | | | |
| Participants | 13a | Describe the flow of participants through the study, including the number of participants with and without the outcome and, if applicable, a summary of the follow-up time. **A diagram may be helpful.**   - Irrelevant, however, the flowchart of the predictive model is found in Figure 4 file | 12 |
|  | 13b | Describe the characteristics of the participants (basic demographics, clinical features, available predictors), including the number of participants with missing data for predictors and outcome.   - irrelevant | - |
| Model development | 14a | Specify the number of participants and outcome events in each analysis.   - Not done in this study | - |
|  | 14b | If done, report the unadjusted association between each candidate predictor and outcome.   - Not done, it’s a predictive model-based artificial neuro-fuzzy approach. | - |
| Model specification | 15a | Present the full prediction model to allow predictions for individuals (i.e., all regression coefficients, and model intercept or baseline survival at a given time point).   - The model description for tracking and projecting PHIVP and PHIVN models begins with six inputs for each model (Figure 2, Equations (1–8)). Appendices III and IV include the final outcome model. RMSE and regression have been used to evaluate the model's performance and correctness. | 9-13 |
|  | 15b | Explain how to the use the prediction model.   - The use of the prediction model can be done by identifying the prediction goal, collecting and processing data, uploading and simulating input data with regression, and interpreting the results. | 8-13 |
| Model performance | 16 | Report performance   - It measures with RMSE and coefficient of determination (regression) for the prediction model found in Table 3 file. | 13 |
| **Discussion** | | | |
| Limitations | 18 | Discuss any limitations of the study (such as nonrepresentative sample, few events per predictor, missing data).   - Huge data requires long simulation time for training model computation; in addition, small numbers of iterations (trails) has a negative impact on the predictor model accuracy | 17 |
| Interpretation | 19b | Give an overall interpretation of the results, considering objectives, limitations, and results from similar studies, and other relevant evidence.   - Provide thorough results together with the predictive model's interpretation, as shown in appendices III and IV (page 2). With a few limitations when compared to other relevant studies, the predictive model satisfies the suggested goals with a satisfactory outcome in the manuscript. | 13-16 |
| Implications | 20 | Discuss the potential clinical use of the model and implications for future research   - Future study by AI developers will create a useful tool to assist PLHIV patients with testing, diagnosis, and HIV+ tracking history, but this will require a patient data center and surveillance system (implications). | 17 |
| **Other information** | | | |
| Supplementary information | 21 | Provide information about the availability of supplementary resources, such as study protocol, Web calculator, and data sets.   - Some data collection is available at <https://www.mnypa.org/docs/1723095739.pdf>. While other surveyed data were used to generate random data for modeling sets, see (**Appendix III, IV**). | 7 |
| Funding | 22 | Funding   - The manuscript has no funding. |  |

We recommend using the TRIPOD Checklist in conjunction with the TRIPOD Explanation and Elaboration document.
