# Supplementary material for: Artificial neuro-fuzzy intelligence for dynamic tracking and prediction of HIV prevalence and new infections among individuals aged 15 to 49 years in Uganda: Computational national-level models
Source: Medicine (Baltimore). 2026 Sep 18;105(38):e50760. doi: 10.1097/MD.0000000000050760 (PMC13593017; doi:10.1097/MD.0000000000050760)
Supplement: Supplementary file 9 [file medi-105-e50760-s009.docx]

Data collection of 146 districts from <https://www.mnypa.org/docs/1723095739.pdf> which are **HHVIP, HHIVN, & ART.**

We select the first 124 highlighted districts for training data and the other 22 districts for testing and validating the predictive tracking models of **PHIVP** and **PHIVN**. The data, such as **FMA**, **INC**, and **EDU**, were collected from the survey, which has been conducted over the past five months. The **SEXR** data ranged between 0 and 1, as estimated from 0.1 (10%) to 1 (100%).

HHIVP = the rates of the historical data of HIV prevalence

HHIVN = the historical data of new HIV incidence

ART = the percentage coverage of antiretroviral therapy

FMA = family member awareness

INC = individual income

EDU = educational level

SEXR = sex rates

Below are data collection with their description

| **District name** | **HHVIP** | **HHIVN** | **ART** | **SEXR** | **FMA** | **INC** | **EDU** |
| --- | --- | --- | --- | --- | --- | --- | --- |
| Abim | 2.6 | 81 | 85.6 | 0.475575 | 0.646174 | 281.697 | 0.765799 |
| Adjumani | 2.4 | 86 | 85.8 | 0.972494 | 0.293714 | 69.76203 | 0.184215 |
| Agago | 6.9 | 250 | 85.5 | 0.203639 | 0.108152 | 24.80801 | 0.838968 |
| Alebtong | 4.3 | 197 | 84 | 0.747253 | 0.252047 | 103.301 | 0.562064 |
| Amolatar | 5.3 | 160 | 81.8 | 0.687295 | 0.907606 | 16.90012 | 0.260341 |
| Amudat | 1.3 | 46 | 48.3 | 0.617069 | 0.541654 | 485.921 | 0.892068 |
| Amuria | 2.9 | 115 | 85.6 | 0.304138 | 0.205324 | 244.9888 | 0.376941 |
| Amuru | 4.8 | 175 | 81.6 | 0.084337 | 0.368863 | 142.9297 | 0.128959 |
| Apac | 6.3 | 258 | 79.3 | 0.50544 | 0.570465 | 205.4767 | 0.876194 |
| Arua | 2.3 | 61 | 83.9 | 0.728117 | 0.048579 | 353.5503 | 0.71452 |
| Arua city | 3.8 | 242 | 88.9 | 0.621689 | 0.82798 | 285.3576 | 0.921971 |
| Budaka | 2.9 | 137 | 77 | 0.151919 | 0.036724 | 498.9448 | 0.676476 |
| Bududa | 1.4 | 35 | 81.6 | 0.657537 | 0.57356 | 154.1387 | 0.391787 |
| Bugiri | 2.4 | 204 | 83.3 | 0.174454 | 0.930891 | 342.7818 | 0.525269 |
| Bugweri | 2.3 | 78 | 80.5 | 0.070861 | 0.503637 | 448.9305 | 0.596247 |
| Buhweju | 3.9 | 102 | 74.9 | 0.310496 | 0.124134 | 462.7287 | 0.878423 |
| Buikwe | 9.1 | 700 | 85.2 | 0.629522 | 0.182206 | 475.5469 | 0.818177 |
| Bukedea | 2.2 | 98 | 86.3 | 0.867093 | 0.573604 | 365.994 | 0.621537 |
| Bukomansimbi | 9.1 | 188 | 87.5 | 0.133847 | 0.421699 | 24.23937 | 0.243944 |
| Bukwo | 1.4 | 34 | 77.2 | 0.248314 | 0.420402 | 233.9545 | 0.384249 |
| Bulambuli | 3.1 | 190 | 55.2 | 0.376016 | 0.722867 | 451.0746 | 0.144224 |
| Buliisa | 3.5 | 118 | 70.6 | 0.739914 | 0.308202 | 48.91463 | 0.211709 |
| Bundibugyo | 2.2 | 115 | 80.3 | 0.584993 | 0.028865 | 470.1265 | 0.809275 |
| Bunyangbu | 9 | 291 | 83.5 | 0.638275 | 0.005493 | 337.5281 | 0.362025 |
| Bushenyi | 9.1 | 362 | 83.6 | 0.63228 | 0.628787 | 322.6375 | 0.805693 |
| Busia | 3.9 | 268 | 83.5 | 0.449877 | 0.915377 | 96.50358 | 0.798566 |
| butaleja | 1.7 | 98 | 77.2 | 0.674922 | 0.766154 | 468.7475 | 0.775318 |
| Butambala | 7.2 | 119 | 82.7 | 0.105633 | 0.507688 | 474.0469 | 0.393749 |
| Butebo | 2.2 | 44 | 80.6 | 0.537351 | 0.012146 | 108.4098 | 0.053287 |
| Buvuma | 4 | 110 | 78 | 0.84181 | 0.765363 | 82.39039 | 0.734677 |
| Buyende | 2.1 | 155 | 80.8 | 0.492238 | 0.638997 | 232.3527 | 0.511219 |
| Dokolo | 4.9 | 187 | 78.2 | 0.85561 | 0.089738 | 319.0179 | 0.625521 |
| Fort portal city | 14.1 | 295 | 84.5 | 0.46764 | 0.152831 | 54.219 | 0.721534 |
| Gomba | 5.8 | 158 | 81.3 | 0.22223 | 0.932036 | 498.1095 | 0.306923 |
| Gulu | 7.2 | 131 | 84 | 0.717052 | 0.701297 | 300.8229 | 0.902759 |
| Gulu city | 10.5 | 460 | 89.8 | 0.029007 | 0.114516 | 436.4899 | 0.935464 |
| Hoima | 4 | 197 | 76.1 | 0.082066 | 0.535809 | 194.818 | 0.714247 |
| Hoima city | 9.6 | 228 | 85.1 | 0.996207 | 0.124854 | 40.94264 | 0.960276 |
| Ibanda | 7.1 | 323 | 80.8 | 0.408917 | 0.311675 | 402.4983 | 0.466589 |
| Iganga | 3.4 | 254 | 82.7 | 0.067722 | 0.772534 | 431.0276 | 0.602862 |
| Isingiro | 4.1 | 395 | 85.3 | 0.544034 | 0.426312 | 243.6725 | 0.743145 |
| Jinja | 3 | 141 | 81.1 | 0.307105 | 0.9902 | 282.2813 | 0.935732 |
| Jinja city | 7.5 | 348 | 83.5 | 0.040215 | 0.216096 | 150.4434 | 0.605341 |
| Kaabong | 0.6 | 15 | 76.8 | 0.606948 | 0.698694 | 300.7686 | 0.716738 |
| Kabale | 5.5 | 227 | 84 | 0.082611 | 0.92508 | 399.1275 | 0.407112 |
| Kabarole | 11.6 | 404 | 85.7 | 0.836056 | 0.186945 | 365.8824 | 0.508757 |
| Kaberamaido | 5 | 123 | 81.1 | 0.630189 | 0.896658 | 151.7839 | 0.203503 |
| Kagadi | 5.4 | 432 | 75.5 | 0.590546 | 0.677971 | 346.413 | 0.235747 |
| Kakumiro | 3.9 | 432 | 74.2 | 0.734838 | 0.911046 | 415.2719 | 0.156536 |
| Kalaki | 3.8 | 87 | 87.4 | 0.436705 | 0.456862 | 58.1756 | 0.785781 |
| Kalangala | 13.1 | 172 | 83.6 | 0.21675 | 0.485432 | 217.5476 | 0.448048 |
| Kaliro | 1.8 | 96 | 75.9 | 0.299323 | 0.219337 | 448.768 | 0.054795 |
| Kalungu | 9.9 | 245 | 87.9 | 0.508619 | 0.971571 | 327.594 | 0.392247 |
| Kampala | 7.4 | 2800 | 84.8 | 0.532051 | 0.667684 | 226.0829 | 0.659348 |
| Kamuli | 2.9 | 263 | 87.1 | 0.523187 | 0.347998 | 326.7956 | 0.050464 |
| Kamwenge | 5 | 300 | 79.6 | 0.506179 | 0.228382 | 258.8879 | 0.102335 |
| Kanungu | 6.6 | 292 | 80.5 | 0.286422 | 0.255669 | 120.3521 | 0.580969 |
| Kapchorwa | 3 | 76 | 68.5 | 0.463596 | 0.342821 | 400.5872 | 0.690301 |
| Kapelebyong | 3.6 | 64 | 82.2 | 0.867709 | 0.136216 | 330.2499 | 0.645229 |
| Karenga | 2.3 | 28 | 78 | 0.69266 | 0.309434 | 363.5387 | 0.936304 |
| Kasese | 2.6 | 383 | 80.9 | 0.63971 | 0.907689 | 371.6239 | 0.883087 |
| Kassanda | 5.5 | 308 | 76.9 | 0.167612 | 0.741077 | 293.4809 | 0.398919 |
| Katakwi | 4.1 | 137 | 83.9 | 0.856308 | 0.206951 | 235.7066 | 0.849351 |
| Kayunga | 5.3 | 336 | 84.6 | 0.239448 | 0.449486 | 420.6337 | 0.557347 |
| Kazo | 4.4 | 171 | 79.2 | 0.088089 | 0.186026 | 360.3291 | 0.052703 |
| Kibaale | 4.4 | 182 | 76.1 | 0.143151 | 0.153817 | 92.4311 | 0.554114 |
| Kiboga | 6.4 | 195 | 84.4 | 0.750968 | 0.046386 | 378.467 | 0.291996 |
| Kibuku | 1.7 | 78 | 80.4 | 0.470786 | 0.129912 | 285.908 | 0.613592 |
| Kikuube | 4.1 | 319 | 70.3 | 0.058327 | 0.940512 | 112.5134 | 0.722333 |
| Kiruhura | 8.9 | 284 | 84.6 | 0.330294 | 0.991309 | 69.23347 | 0.535081 |
| Kiryandongo | 4.2 | 253 | 79 | 0.299011 | 0.176459 | 289.9018 | 0.332872 |
| Kisoro | 2.2 | 117 | 81.6 | 0.930104 | 0.631189 | 200.0924 | 0.220163 |
| Kitagwenda | 5.4 | 166 | 78.4 | 0.353984 | 0.853548 | 273.1384 | 0.267932 |
| Kitgum | 5.3 | 190 | 88.3 | 0.228188 | 0.724856 | 66.27275 | 0.883398 |
| Koboko | 1.2 | 62 | 84 | 0.504396 | 0.150387 | 448.2073 | 0.487939 |
| Kole | 5.2 | 255 | 82.7 | 0.296342 | 0.797317 | 368.5445 | 0.467096 |
| Kotido | 0.9 | 35 | 78.5 | 0.268701 | 0.342364 | 15.63423 | 0.050429 |
| Kumi | 2.3 | 109 | 84.3 | 0.989122 | 0.253523 | 166.4049 | 0.735842 |
| Kwania | 5.9 | 221 | 80.7 | 0.389578 | 0.702351 | 265.3647 | 0.035184 |
| Kween | 2.2 | 58 | 56 | 0.84856 | 0.420471 | 133.6995 | 0.452868 |
| Kyankwanzi | 4.6 | 257 | 79.1 | 0.949638 | 0.203841 | 349.4516 | 0.964728 |
| Kyegewa | 3.6 | 367 | 74.3 | 0.528773 | 0.792869 | 141.0509 | 0.41773 |
| Kyenjojo | 6.7 | 600 | 81.8 | 0.045252 | 0.351551 | 62.72681 | 0.388406 |
| Kyotera | 12.2 | 450 | 89.5 | 0.055163 | 0.292556 | 338.1698 | 0.365891 |
| Lamwo | 5.9 | 128 | 84.7 | 0.49033 | 0.352459 | 282.3605 | 0.960515 |
| Lira | 5.7 | 225 | 82.7 | 0.768568 | 0.664081 | 304.2929 | 0.152625 |
| Lira city | 12 | 600 | 86.9 | 0.740579 | 0.463911 | 465.0921 | 0.38554 |
| Luuka | 2.2 | 111 | 70.9 | 0.31274 | 0.343484 | 155.4241 | 0.882309 |
| Luwero | 6.7 | 600 | 85.1 | 0.373451 | 0.306196 | 256.9895 | 0.364514 |
| Lwengo | 8.4 | 314 | 84.7 | 0.625319 | 0.491916 | 93.65021 | 0.221783 |
| Lyantonde | 8.4 | 160 | 84.4 | 0.189428 | 0.153174 | 431.3459 | 0.306773 |
| Madi Akollo | 1.9 | 56 | 79.8 | 0.80627 | 0.313197 | 10.52521 | 0.881333 |
| Manafwa | 1.8 | 57 | 75.6 | 0.492681 | 0.331595 | 194.6351 | 0.252587 |
| Maracha | 1.1 | 35 | 82.5 | 0.701421 | 0.099655 | 113.1121 | 0.467716 |
| Masaka | 9.2 | 156 | 86.5 | 0.020688 | 0.377709 | 323.1819 | 0.827615 |
| Masaka city | 11.3 | 437 | 86.4 | 0.323062 | 0.948912 | 22.98363 | 0.632573 |
| Masindi | 6.1 | 390 | 80.2 | 0.373923 | 0.502128 | 262.5826 | 0.702559 |
| Mayuge | 2.6 | 268 | 80.7 | 0.692176 | 0.194393 | 33.8927 | 0.254538 |
| Mbale | 3.3 | 150 | 79.5 | 0.73266 | 0.922893 | 70.34464 | 0.112821 |
| Mbale city | 4.8 | 319 | 86.8 | 0.525286 | 0.540889 | 467.5387 | 0.850705 |
| Mbarara | 7.8 | 201 | 86.3 | 0.511788 | 0.423916 | 294.1109 | 0.464464 |
| Mbarara city | 11.1 | 491 | 84.1 | 0.66242 | 0.926296 | 165.5026 | 0.59371 |
| Mitooma | 5.4 | 158 | 83 | 0.617433 | 0.111599 | 127.6159 | 0.805589 |
| Mityana | 7.3 | 452 | 82.8 | 0.181179 | 0.748316 | 240.5529 | 0.190125 |
| Moroto | 1.8 | 41 | 75.1 | 0.967882 | 0.46386 | 285.1077 | 0.248455 |
| Moyo | 2.4 | 44 | 82.9 | 0.306445 | 0.126769 | 334.8906 | 0.359451 |
| Mpigi | 8.2 | 405 | 82.7 | 0.17953 | 0.054238 | 64.6727 | 0.944635 |
| Mubende | 4.9 | 600 | 82.1 | 0.07808 | 0.918287 | 145.9092 | 0.122997 |
| Mukono | 5.3 | 700 | 82.7 | 0.713934 | 0.817488 | 473.0141 | 0.293278 |
| Nabilatuk | 0.5 | 9 | 85.2 | 0.280269 | 0.831497 | 407.3538 | 0.010165 |
| Nakapiripirit | 1.4 | 30 | 81.9 | 0.001987 | 0.237515 | 461.3514 | 0.894346 |
| Nakaseke | 4.9 | 205 | 82.9 | 0.935009 | 0.898354 | 182.1693 | 0.75988 |
| Nakasongola | 4.6 | 177 | 84.7 | 0.45577 | 0.894299 | 49.05163 | 0.067015 |
| Namayingo | 4.8 | 178 | 88.9 | 0.359024 | 0.965183 | 33.74021 | 0.176872 |
| Namisindwa | 1.5 | 62 | 76.4 | 0.522008 | 0.987772 | 92.05405 | 0.01005 |
| Namutumba | 1.9 | 102 | 83.4 | 0.315842 | 0.076886 | 342.3952 | 0.402855 |
| Napak | 1.2 | 32 | 81.2 | 0.224779 | 0.988174 | 376.7538 | 0.524252 |
| Nebbi | 3.8 | 184 | 81 | 0.756583 | 0.223691 | 281.4722 | 0.453579 |
| Ngora | 2.3 | 64 | 86.7 | 0.547666 | 0.055489 | 387.701 | 0.20001 |
| Ntoroko | 4.1 | 61 | 79.4 | 0.359982 | 0.285655 | 119.5778 | 0.386866 |
| Ntungamo | 6.4 | 600 | 80.6 | 0.059347 | 0.554411 | 449.2029 | 0.603114 |
| Nwoya | 4.4 | 218 | 86.2 | 0.59545 | 0.900166 | 411.4294 | 0.045024 |
| Obongi | 1.7 | 14 | 85.2 | 0.029263 | 0.789803 | 317.2213 | 0.201806 |
| Omoro | 7.8 | 216 | 86.7 | 0.313394 | 0.103673 | 282.8034 | 0.037546 |
| Otuke | 3.9 | 89 | 87.7 | 0.036949 | 0.136042 | 478.8372 | 0.237575 |
| Oyam | 5.5 | 438 | 79 | 0.81029 | 0.687064 | 21.3564 | 0.124211 |
| Pader | 7.3 | 226 | 85.3 | 0.771161 | 0.933358 | 350.383 | 0.071691 |
| Pakwach | 3.5 | 124 | 82 | 0.115599 | 0.88084 | 340.9025 | 0.068068 |
| Pallisa | 1.1 | 78 | 80.1 | 0.417268 | 0.1826 | 495.4127 | 0.06037 |
| Rakai | 5.2 | 247 | 87.6 | 0.087784 | 0.298198 | 70.11733 | 0.797192 |
| Rubanda | 2.9 | 98 | 79.6 | 0.696527 | 0.479432 | 429.4165 | 0.848321 |
| Rubirizi | 6.7 | 145 | 83.7 | 0.356124 | 0.537876 | 36.09681 | 0.85239 |
| Rukiga | 5 | 81 | 81.8 | 0.635111 | 0.363561 | 241.9767 | 0.950948 |
| Rukungiri | 8.3 | 412 | 84.2 | 0.566798 | 0.032703 | 51.39908 | 0.902535 |
| Rwampara | 7.8 | 177 | 84.6 | 0.268198 | 0.310116 | 197.3773 | 0.478681 |
| Serere | 2 | 124 | 86.7 | 0.737006 | 0.306386 | 383.7941 | 0.323569 |
| Sheema | 2.8 | 253 | 85.2 | 0.476947 | 0.929458 | 229.3061 | 0.39977 |
| Sironko | 2.8 | 129 | 82 | 0.330482 | 0.81159 | 273.9538 | 0.360007 |
| Soroti | 2.8 | 113 | 85 | 0.049855 | 0.44921 | 172.2051 | 0.235075 |
| Soroti city | 12.5 | 294 | 86.9 | 0.510766 | 0.434455 | 464.3639 | 0.978406 |
| Ssembabule | 6.5 | 306 | 87.7 | 0.25827 | 0.286982 | 492.7203 | 0.47087 |
| Terego | 1.4 | 56 | 86.3 | 0.24729 | 0.187127 | 367.0784 | 0.006725 |
| Tororo | 5.1 | 482 | 87.7 | 0.089388 | 0.108442 | 212.25 | 0.112842 |
| Wakiso | 7.2 | 4900 | 86.2 | 0.247193 | 0.604845 | 265.9218 | 0.907129 |
| Yumbe | 1.1 | 121 | 88.4 | 0.957376 | 0.749176 | 15.12202 | 0.85206 |
| Zombo | 3.7 | 166 | 80.7 | 0.569301 | 0.426341 | 284.0702 | 0.794823 |
